# Grapevine Rootstocks Differentially Affect the Rate of Ripening and Modulate Auxin-Related Genes in Cabernet Sauvignon Berries

**DOI:** 10.3389/fpls.2016.00069

**Published:** 2016-02-09

**Authors:** Massimiliano Corso, Alessandro Vannozzi, Fiorenza Ziliotto, Mohamed Zouine, Elie Maza, Tommaso Nicolato, Nicola Vitulo, Franco Meggio, Giorgio Valle, Mondher Bouzayen, Maren Müller, Sergi Munné-Bosch, Margherita Lucchin, Claudio Bonghi

**Affiliations:** ^1^Department of Agronomy, Food, Natural resources, Animals and Environment, University of Padova AgripolisLegnaro, Italy; ^2^Centro Interdipartimentale per la Ricerca in Viticoltura e Enologia, University of PadovaConegliano, Italy; ^3^Genomics and Biotechnology of Fruit Laboratory, Institut National Polytechnique de ToulouseToulouse, France; ^4^Centro di Ricerca Interdipartimentale per le Biotecnologie Innovative, University of PadovaPadova, Italy; ^5^Department of Vegetal Biology, University of BarcelonaBarcelona, Spain

**Keywords:** fruit development, polyphenols biosynthesis, auxin conjugation, transcriptional program, grapevine

## Abstract

In modern viticulture, grafting commercial grapevine varieties on interspecific rootstocks is a common practice required for conferring resistance to many biotic and abiotic stresses. Nevertheless, the use of rootstocks to gain these essential traits is also known to impact grape berry development and quality, although the underlying mechanisms are still poorly understood. In grape berries, the onset of ripening (véraison) is regulated by a complex network of mobile signals including hormones such as auxins, ethylene, abscisic acid, and brassinosteroids. Recently, a new rootstock, designated M4, was selected based on its enhanced tolerance to water stress and medium vigor. This study investigates the effect of M4 on Cabernet Sauvignon (CS) berry development in comparison to the commercial 1103P rootstock. Physical and biochemical parameters showed that the ripening rate of CS berries is faster when grafted onto M4. A multifactorial analysis performed on mRNA-Seq data obtained from skin and pulp of berries grown in both graft combinations revealed that genes controlling auxin action (*ARF* and *Aux/IAA*) represent one of main categories affected by the rootstock genotype. Considering that the level of auxin tightly regulates the transcription of these genes, we investigated the behavior of the main gene families involved in auxin biosynthesis and conjugation. Molecular and biochemical analyses confirmed a link between the rate of berry development and the modulation of auxin metabolism. Moreover, the data indicate that this phenomenon appears to be particularly pronounced in skin tissue in comparison to the flesh.

## Introduction

In Europe, *Vitis vinifera* varieties are grown as scion grafted onto a rootstock. At first, grafting was adopted with the aim of preventing devastation to European viticulture by Phylloxera. This gradually imposed the use of rootstocks as general practice and the development of new rootstock genotypes became an important issue in modern viticulture (Whiting, 2004). The use of rootstocks was proved to be beneficial in terms of adaptation to different soil types and to biotic (e.g., soil borne pests) and abiotic (e.g., salinity, water or oxygen deficit) factors (Marguerit et al., 2012; Tramontini et al., 2013; Corso et al., 2015). Rootstocks can also be used to confer other advantages affecting physiological processes at the scion level, such as biomass accumulation, quality yields, vine vigor, and grape berry quality (Walker et al., 2000; Gregory et al., 2013; Berdeja et al., 2015). The beneficial effects of rootstocks on stress resistance and vegetative growth represent an extremely important issue in viticulture, but their effect on grape development rates and on berry quality also warrants investigation. Although it is widely known that the rootstock influences grapevine reproductive performance and berry development (Kidman et al., 2013), studies specifically addressing the relationship between a given graft combination and the berry ripening evolution are still lacking.

Grape berry development exhibits a double-sigmoid pattern characterized by two phases of rapid growth separated by a lag phase during which little or no growth occurs (Coombe and McCarthy, 2000). Several hormones participate in the control of grape berry development and ripening, such as auxin (IAA), ethylene, abscisic acid (ABA), gibberellins (GAs), cytokinins (CKs), and brassinosteroids (BRs) (Davies and Böttcher, 2009). The early stages of berry development, from fertilization to fruit set, are mainly driven by IAA, CKs, and GAs to promote cell division and cell expansion. Although these hormones have a pivotal role in berry development, they are mostly produced by the seeds (Giribaldi et al., 2010). Thereafter, the changes occurring from pre-véraison to full ripening are associated with sequential increases in ethylene, brassinosteroids, and ABA content (Kuhn et al., 2013). Exogenous applications of hormones positively modulate many ripening-related processes such as anthocyanin accumulation and the uptake/storage of sugars in berries, via the re-programming of gene expression (Chervin et al., 2008; Giribaldi et al., 2010; Böttcher et al., 2011; Ziliotto et al., 2012). In particular, exogenous application of auxin and its analogs at pre-véraison stage causes a shift in ripening and a repression of several ripening-related genes (Davies et al., 1997; Böttcher et al., 2011; Ziliotto et al., 2012). Based on these observations it has been postulated that a decrease in IAA content is necessary to trigger the onset of ripening (Deluc et al., 2007; Ziliotto et al., 2012). This hypothesis has been confirmed by the observation that berries with a slow ripening progression have a high seed-to-berry weight ratio associated with high auxin and low ABA content (Gouthu and Deluc, 2015). Böttcher et al. (2010) speculated that in grapevine, the auxin decrease and maintenance of low IAA active forms may be due to their conjugation with amino acids, mediated by the auxin-responsive Gretchen Hagen 3 (GH3) proteins. However, to further understanding into the role of auxin in fruit development and ripening it is necessary to consider not only the hormone concentration but also the downstream signaling events. Auxin signaling is initiated through binding of the hormone to the Transport Inhibitor Response1/Auxin Signaling F-Box protein (TIR1/AFB) and Auxin/Indole Acetic Acid (Aux/IAA) protein co-receptors, which results in the targeting of Aux/IAA proteins for degradation. The degradation of Aux/IAA proteins allows the release of Auxin Response Factors (ARF), the transcription factors that regulate the expression of auxin-responsive genes. The expression of *Aux/IAAs* and *ARFs* during fruit development and ripening has been extensively studied in many species (Pattison et al., 2014) and in particular in those bearing fleshy fruits (Audran-Delalande et al., 2012; Zouine et al., 2014), although to a lesser extent in grapevine (Çakir et al., 2013; Wan et al., 2014).

Recently, it was demonstrated that grafting the same scion on different rootstock induces extensive transcriptional re-programming in the shoot apex and in berries, particularly for genes involved in hormone signaling (Cookson and Ollat, 2013; Berdeja et al., 2015). This observation is in agreement with the hypothesis for a role of rootstock in the control of scion growth and reproductive activity by the modulation of hormone signaling pathways (Gregory et al., 2013). In order to clarify this aspect, we conducted a physical, biochemical, and transcriptional analysis on berries obtained from *V. vinifera* cv Cabernet Sauvignon (CS) plants grafted onto M4, a rootstock characterized by high tolerance to stress and medium vigor (Meggio et al., 2014), and 1103 Paulsen (1103P), a vigorous commercial rootstock. Data indicated that the ripening rate in berries of CS grafted onto M4 (CS/M4) was faster than those grown on CS/1103P combination. To investigate the relationship existing between the rootstock and the ripening rate in both the graft combinations, we analyzed the berry transcriptome during development and ripening by mean of mRNA-Seq analysis. Molecular analyses indicated that grafting the same variety (CS) on different rootstocks (1103P and M4) alters the expression of several genes, including those belonging to the main multigenic families involved in auxin biosynthesis, conjugation, and action and, consequently, the auxin levels in skin and flesh. A possible consequence of this alteration is a change in the berry ripening rate. This phenomenon was more pronounced in the berry skin in comparison to the flesh.

## Materials and methods

### Plant material, experimental design and meteorological data

Sampling was performed in 2011 and 2012 on *V.vinifera* cv Cabernet Sauvignon plants grafted onto 1103P (*V. berlandieri* × *V. rupestris*) and M4 [(*V. vinifera* × *V. berlandieri*) × *V. berlandieri* × cv Resseguier n.1] rootstocks located in Verona, Italy (Novaglie, 45°28′42.4°N, 11°02′40.4°E; Pasqua vigneti e cantine) and grown from 2003 in open field on a clay-calcareous soil. All vines were of the same age and were grafted in 2002. The two graft combinations were growth in adjacent rows, with north–south orientation. Meteorological data, were registered from the meteorological station of Grezzana (45° 30′ 35.22″ N, 11° 00′ 48.54″ E, 156 m a. s. l.) and collected by the Regional Agency for the Environmental Protection of Veneto (ARPAV), Italy. The dataset consisted of meteorological time series from January 1992 to December 2012, a series of 20 years that enabled climatological study to be performed. Meteorological data used for the purposes of the study consisted of precipitations (mm) and air temperature (°C) measured at 2 m.

During both the growing seasons (2011 and 2012), berries grown on CS/1103P and CS/M4 graft combinations were sampled at five developmental stages following the modified Eichhorn and Lorenz (E-L) developmental scale proposed by Coombe (1995).

**Figure 1:**
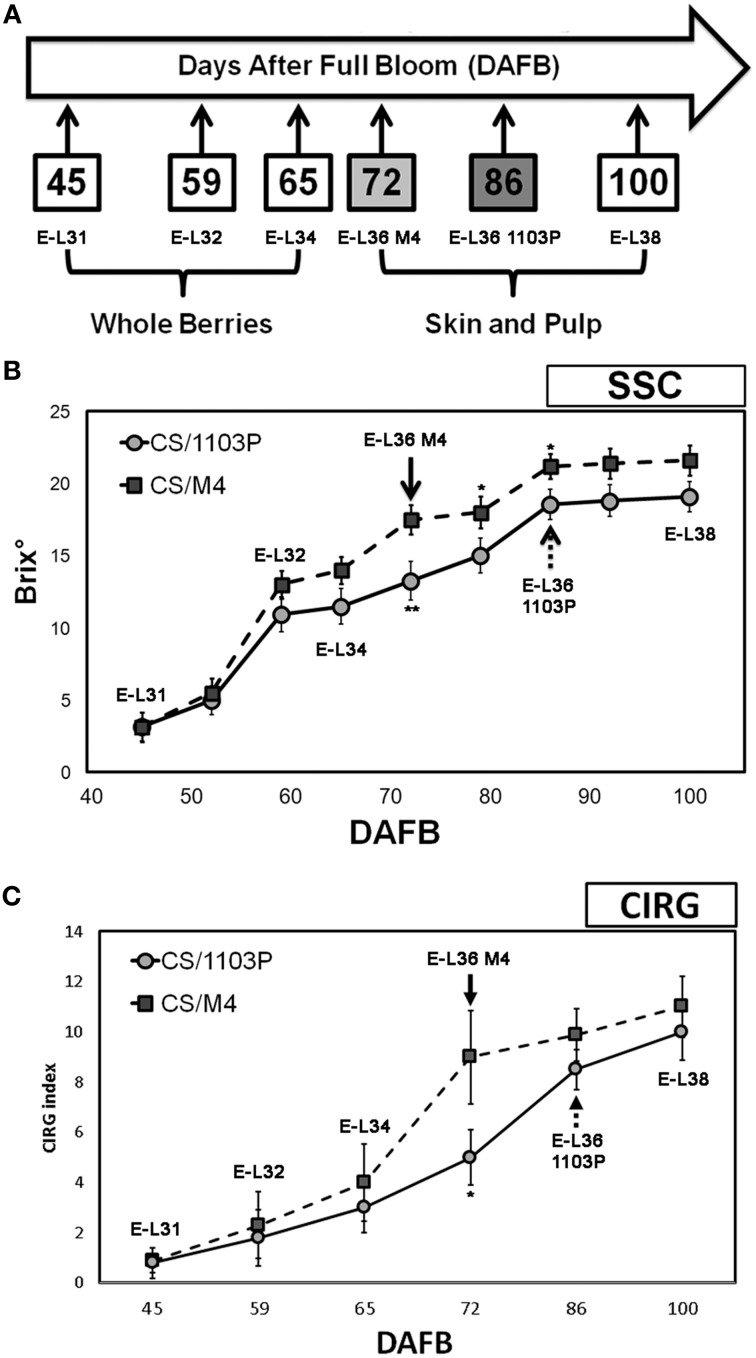
**(A)** Schematic representation of the experimental trial. Samplings of berries grown in both 1103P/CS and M4/CS graft combinations were performed at 45 (E-L31), 59 (E-L32), 65 (E-L34), 72 (E-L36 M4), 86 (E-L36 1103P), and 100 (E-L38) DAFB. **(B)** Soluble solids content in CS/M4 (squares) and CS/1103P (circles) throughout fruit development. **(C)** CIRG values of CS/M4 (square) and CS/1103P (circle) graft combinations throughout fruit development. Bars represent the SD of 100 berries. CS/M4 and CS/1103P data from samples collected at the same DAFB were statistically treated using Student's *t*-test (^*^P < 0.05; ^**^P < 0.01).

The evolution of berry development and ripening on both graft combinations was monitored by measuring the sugar content and the skin pigmentation. Total Soluble Solids and colorimetric analysis were determined in 100 berries collected from 10 different plants (one bunch per plant) for each time point and graft combination considered in the experiment. Color analyses were carried out with a CR-10 colorimeter (Konica-Minolta Holdings Inc., Tokyo, Japan) based on the L^*^a^*^b^*^ space, the defining brightness (L^*^, from white to black), and the chromatic coordinates (a^*^, from red to green; b^*^, from yellow to blue). Other parameters, such as hue angle (h), chroma (C), and Color Index for red Grapes (CIRG) were also calculated according to Carreño et al. (1995). At pre-véraison stages (E-L31, E-L32, and E-L34) the whole berries were considered, whereas at E-L36 and E-L38 stages, skin and pulp were sampled separately (Figure 1A).

Samplings at E-L36 stage were performed at 72 DAFB (Days After Full Bloom) for CS/M4 and 86 DAFB for CS/1103P when berries showed similar sugar content and skin color, whereas samplings at E-L31, E-L32, E-L34, and E-L38 corresponded to similar date in the two graft combinations and precisely to 45, 59, 65, and 100 DAFB (Figure 1A). In 2012 berries were collected at the same E-L stages considered in 2011. Two biological replicates, sampled in 2011, were used for mRNA-Seq, while three biological replicates, sampled in 2012, were considered for quantitative RT-PCR (qRT-PCR). Each replicate was composed of 100 berries sampled from 50 different bunches (two berries collected from the median position of each cluster) according to the CIRG.

### RNA-Seq and quantitative RT-PCR analyses

Total RNA for transcriptome sequencing was extracted from samples collected at E-L31, E-L36, and E-L38 using the perchlorate method as reported by Ziliotto et al. (2012). Poly (A) mRNA was purified from total RNA using the Dynabeads “mRNA direct kit” (Invitrogen pn 610.12). Samples for Ligation Sequencing were prepared according to the SOLiD Whole transcriptome library preparation protocol (pn 4452437 Rev. B). Reads were aligned to the reference grape genome using PASS aligner (Campagna et al., 2009). The percentage identity was set to 90% with one gap allowed whereas the quality filtering parameters were set automatically by PASS. Moreover, a minimum reads length cut-off of 50 and 30 nt was set for the forward sequences and reverse reads, respectively. The spliced reads were identified using the procedure described in PASS manual (http://pass.cribi.unipd.it). Forward and reverse reads were aligned independently on the reference genome. PASS-pair was used from the PASS package to perform the pairing between forward and reverse reads and to select only those sequences that are uniquely aligned. The version 1 (V1) of grape gene prediction (http://genomes.cribi.unipd.it/grape) was used as a reference genome, whereas htseq-counts program (http://www-huber.embl.de/users/anders/HTSeq/doc/count.html) was adopted to quantify gene transcripts abundance. Gene expression data have been submitted to Gene Expression Omnibus (GEO) (accession no. SRA110619) at the NCBI (https://www.ncbi.nlm.nih.gov/geo/). Quantitative RT-PCRs (qPCR) were performed as described in Ziliotto et al. (2012). Gene specific primers are listed in Supplementary Table S1.

### Statistical and bioinformatics analysis

*DEseq* R package (http://www.r-project.org) was used to perform the statistical analyses for discovering differentially expressed genes (DEGs). In order to evaluate the single effects of the rootstock (R: 1103P and M4), tissue (T: whole berries, skin and pulp), and phenological phase (PP: E-L31, pre-véraison; E-L36, mid/late véraison; E-L38, ripening) on gene expression, a multifactorial analysis was conducted using the multi-factor designs method of *DEseq* (Anders and Huber, 2010) (http://bioconductor.org/packages/release/bioc/html/DESeq.html). This method evaluates the weight of each factor considered in the analysis (R, T, and P) and its impact on DEGs, according to a false discovery rate (FDR) corrected *p*-value lower than 0.05.Enrichment analysis was performed for each set of DEGs (R, T, and P) by using BiNGO tool (Maere et al., 2005) with the built-in Fisher's exact test function and an FDR corrected *p*-value lower than 0.05. Hierarchical clustering analysis on mRNA-Seq data was carried out using Multi Experiment Viewer software (MeV; http://www.tm4.org; Saeed et al., 2006). Expression values used for the analysis were filtered based on the 5% of their median. Principal Component Analysis (PCA) and related graphs were carried out using “prcomp” and “scatterplot3d” R packages, respectively.

### LC-ESI-MS/MS analysis of IAA and IAA-Asp in CS/M4 and CS/1103P berries

The samples processed for the mRNA-Seq analysis at E-L31, E-L36, and E-L38, together with those collected at E-L32 and E-L34, were also used for LC-MS/MS quantification. IAA and IAA-Asp were extracted and quantified from 100 mg of tissue as described by Müller and Munné-Bosch (2011) with some modifications. Sample tissue was spiked with [^2^H_5_]IAA and [^2^H_5_]IAA-Asp as internal standards and then extracted with 0.2 ml methanol, isopropanol, and glacial acetic acid (20:79:1, v/v/v) using ultra sonication (4–7°C). After centrifugation (14,000 × g for 15 min at 4°C), the supernatant was collected and the pellet re-extracted with 0.2 ml of extraction solvent. Then, the supernatants were combined, centrifuged (14,000 × g for 5 min at 4°C) and filtered through a 0.22 μm PTFE filter to be analyzed with an UPLC/ESI-MS/MS system. The LC system consisted of an Aquity UPLCTM System (Waters, Milford, MA USA) and samples (5 μl) were first separated on a C18 Kinetex column (50 × 2.1 mm, 1.7 μm; Phenomenex, Macclesfield, UK) using the following solvent conditions: 0–4 min linear gradient from 99% of solvent A to 1%, held for 0.2 min, from 1% of solvent A to 99% in 0.2 min and held for 0.6 min. Gradient solvents consisted of water and 0.05% glacial acetic acid (solvent A) and acetronitrile with 0.05% glacial acetic acid (solvent B). MS/MS experiments and detection were performed on an API 3000 triple quadruple mass spectrometer (PE Sciex, Concord, Ont., Canada) by multiple reactions monitoring (MRM) in negative ion mode. The optimized MS/MS conditions were determined in infusion experiments using purified IAA and IAA-Asp and their isotopical labeled internal standards. MRM transitions were 174/130 for IAA and 179/135 for [^2^H_5_]-IAA with the collision energy (CE) of −15 eV and collision cell exit potential (CXP) of −15 eV. MRM transition of IAA-Asp was 289/88 and 294/89 for [^2^H_5_] IAA-Asp with CE of −36 eV and CXP of −15 eV. IAA and IAA-Asp quantification were performed by a ten-point calibration curve including [^2^H_5_]IAA and [^2^H_5_]IAA-Asp as internal standards using Analyst™ software (PE Sciex, Concord, Ont., Canada). The data were subjected to a Duncan's multiple-range test, performed using “agricolae” R package.

## Results

### Biochemical and colorimetric analyses showed different berry ripening evolution in CS/M4 and CS/1103P graft combinations

Berries grown on CS/1103P and CS/M4 graft combinations were sampled at five developmental stages following the modified Eichhorn and Lorenz developmental scale proposed by Coombe (1995). The criteria used for evaluating the evolution of grape development and ripening were the measurement of sugar content (SSC) and the CIRG values (Figure 1). Developmental stages considered in the study were defined as follows: (a) E-L 31: small hard green berries accumulating organic acids; (b) E-L 32: beginning of bunch closure, berries tight at touch; (c) E-L 34: stage immediately preceding véraison (onset of ripening) characterized by green berries; E-L 36: sugar (15–18°Brix) and anthocyanins accumulation and active growth due to cell enlargement (mid/late véraison) (Fortes et al., 2011) and E-L 38: harvest time. Biochemical and physical measurements performed on berries during 2011 growing season indicated different rate of berry development (Figure 1). The pre-véraison stages (E-L31-34) were reached almost simultaneously by berries grown in both graft combinations, as indicated by the similar evolution of SSC and CIRG (Figures 1B,C), while the ripening rate was different. In fact, the SSC (17.1°Brix ± 1.5) and CIRG (8.2 ± 2.1) values showed by CS/M4 berries at 72 DAFB (E-L36) were reached by CS/1103P berries at 86 DAFB. At harvest (E-L38) berries from both graft combinations had similar SSC values suggesting a recovery of the CS/1103P combination respect to CS/M4. Similar results were obtained by analysing the skin color evolution. Based on CIRG index values, the pigmentation of berry skin in CS/1103P displayed a 14-days delay compared to CS/M4, while at harvest (E-L38) berries from both graft combinations reached the same CIRG value, confirming a recovery from CS/1103P (Figure 1C, Supplementary Figure S1). The different evolution of berry development and ripening in CS/1103P and CS/M4 berries was observed also in 2012 (Supplementary Figure S2), although the two growing seasons were characterized by significant differences in temperature excursions as described in Supplementary Data S1.

### Whole transcriptome analysis revealed that M4 and 1103P differently modulate the expression of auxin-related genes in CS berries

**Figure 2:**
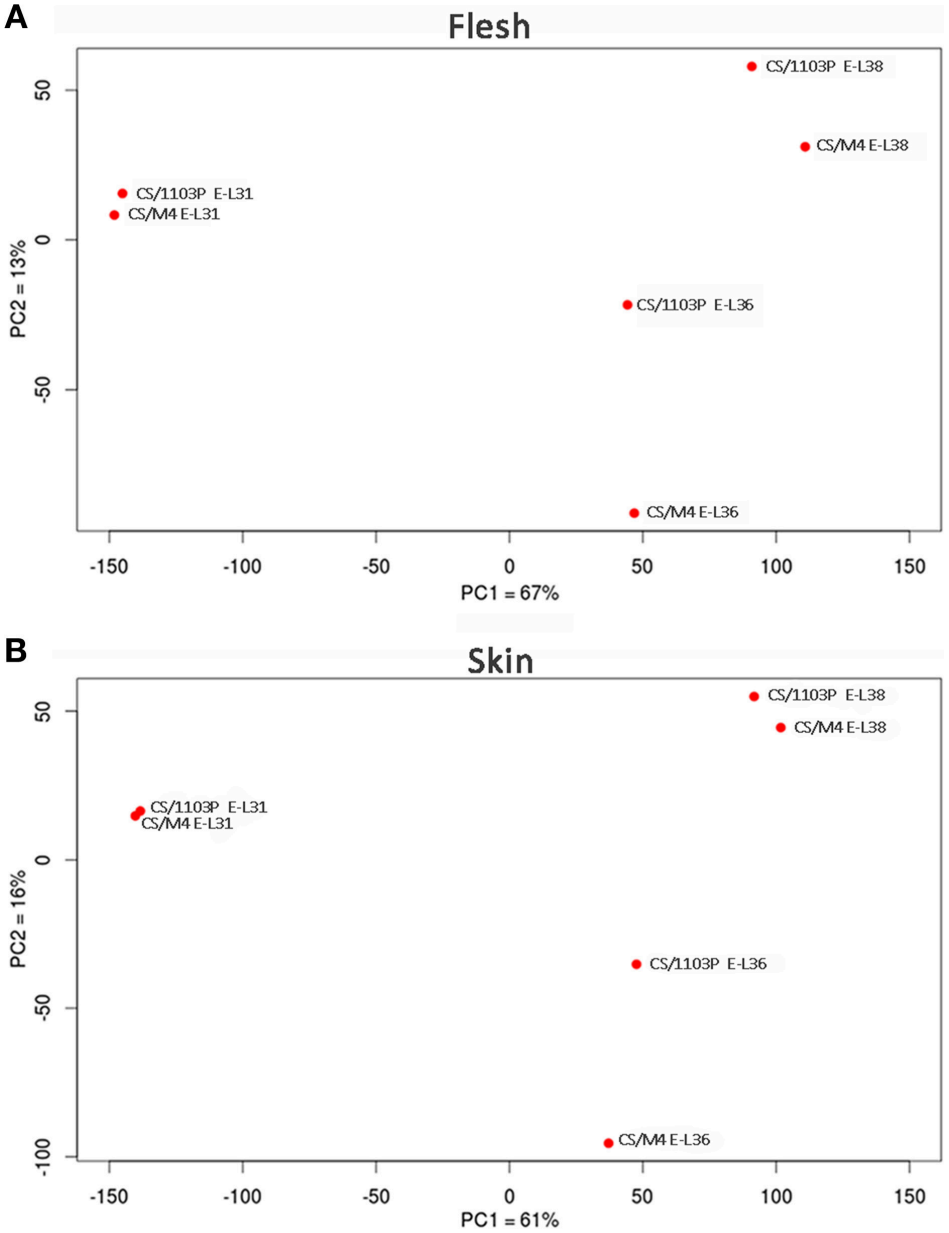
**Bidimensional PCA plot of row transcriptome data**. Cabernet Sauvignon (CS) grafted onto 1103P (CS/1103P) and M4 (CS/M4) samples distribution according to PC1 and PC2. Two separate PCAs were carried out for flesh **(A)** and skin **(B)** mRNA-seq data. Percent of variance is also reported for each component on the corresponding axes.

In order to confirm from a molecular point of view the delay observed in ripening rate between the two graft combinations, we performed a comparative mRNA-Seq transcriptome profiling on CS/M4 and CS/1103P berries collected at E-L31, E-L36, and E-L38. Approximately 2 billion paired-end reads (75 and 35 bp length for forward and reverse reads, respectively) were produced, with a total number of reads for each sample ranging from 36 to 65 million and a median of 52 million reads (Supplementary Table S2). On average, 91% of the reads passed the quality control test (filter based on read length after trimming the low quality bases) and were mapped to the PN40024 12X V1 grape reference genome (Jaillon et al., 2007; http://genomes.cribi.unipd.it/grape), producing approximately 20–42 million unique mapping reads depending on the sample considered. The rate of read mapping on known genes was on average 87% and the number of predicted genes covered at least by five independent reads was approximately 63% (Supplementary Table S2). A PCA performed on mRNA-Seq counts normalized and filtered (*n* > 10) confirmed that both in skin and flesh the transcriptome of CS/1103P and CS/M4 berries collected at E-L31 and E-L38 clustered together, as well as those of berries sampled at E-L36, although sampled at different DAFB (Figure 2).

**Figure 3:**
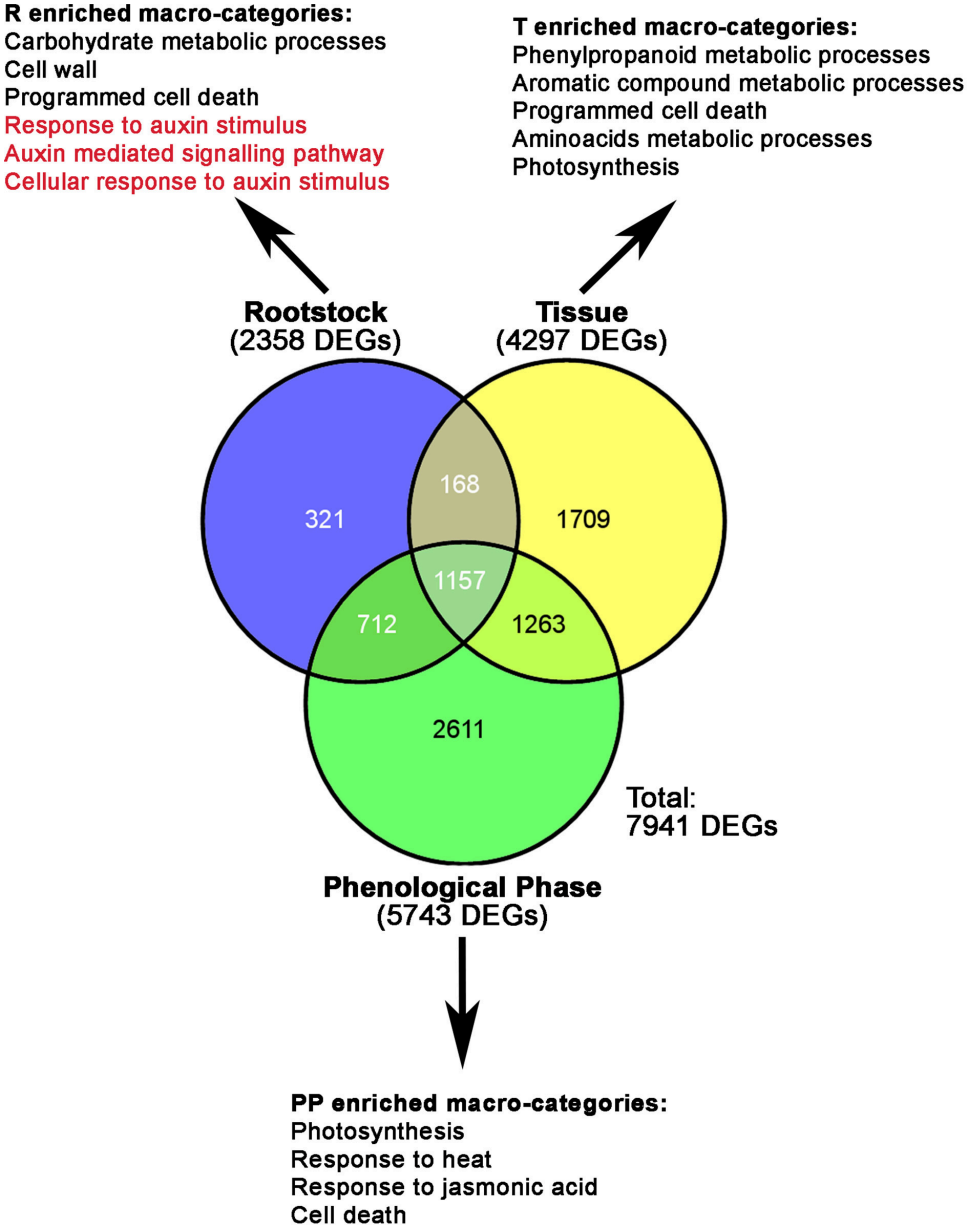
**Venn diagrams showing the relative numbers of specific and common DEGs resulting from the multifactorial analyses, according to an FDR corrected *p*-value lower than 0.05**. Numbers of DEGs influenced by each component (R, Rootstock; T, Tissue; PP, Phenological Phase) are given in brackets. The most significant categories resulted from GO enrichment analysis carried out on DEGs influenced by R, T, and P, are also indicated.

A multifactorial statistical analysis on mRNA-Seq data was performed to identify those genes whose expression is influenced by the effects of three factors: the rootstock (R, M4, or 1103P); the tissue type (T, whole berry, skin, or pulp) and the phenological phase (PP, E-L31, E-L36, or E-L38), on the transcriptome responses. The singular (R, T, P) impact of each component on genes expression was calculated according to a FDR corrected *p*-value lower than 0.05. A complete list of DEGs whose expression is influenced by these factors is reported in Supplementary Table S3, while Figure 3 provides a graphical representation of the total amount of DEGs influenced by each single component. Amongst these, 2358 genes were differentially expressed due to different rootstocks. Expanding the comparison to include different tissue types revealed 4297 genes showed differential expression. The majority of DEGs were influenced by the phenological phase, with 5743 transcripts showing altered expression. In order to identify specific metabolic pathways differentially regulated by M4 and 1103P rootstocks in CS berries, DEGs resulting from multifactorial analysis were associated to their respective GO terms, and a GO enrichment analysis was carried out for each dataset (Figure 3 and Supplementary Table S4). Enriched GO terms associated with metabolic and physiological processes (i.e., photosynthesis, carbohydrate metabolism, aromatic compound metabolism, and phenylpropanoid metabolism) were identified amongst those DEGs affected by either single or combined factors, whereas GO terms related to regulatory mechanisms such as hormone metabolism and action were overrepresented only in those DEGs influenced by a single factor. Amongst these we considered of particular interest were the categories related to response to auxin stimulus (GO: 9733), auxin mediated signaling pathway (GO: 9734), and cellular response to auxin stimulus (GO: 71365), not only because of the role of this hormone in grape berry development, but also because the expression of genes belonging to these categories was affected exclusively by the rootstock (Figure 3 and Supplementary Table S4).

A large number of genes belonging to these auxin-related GO categories were found to encode for Auxin/Indole-3-Acetic Acid (Aux/IAA) and the Auxin Response (ARF) transcription factors, representing two key families of auxin-response regulators (Supplementary Tables S4, S5). Recently, Çakir et al. (2013) and Wan et al. (2014) performed a genomic characterization of both the *ARF* and *Aux/IAA* gene families in grapevine. In the current study we proposed and used a new classification of both gene families (together with the *GH3*), based on the grapevine gene nomenclature system developed by Grimplet et al. (2014), as illustrated in Supplementary Results S1.

**Figure 4:**
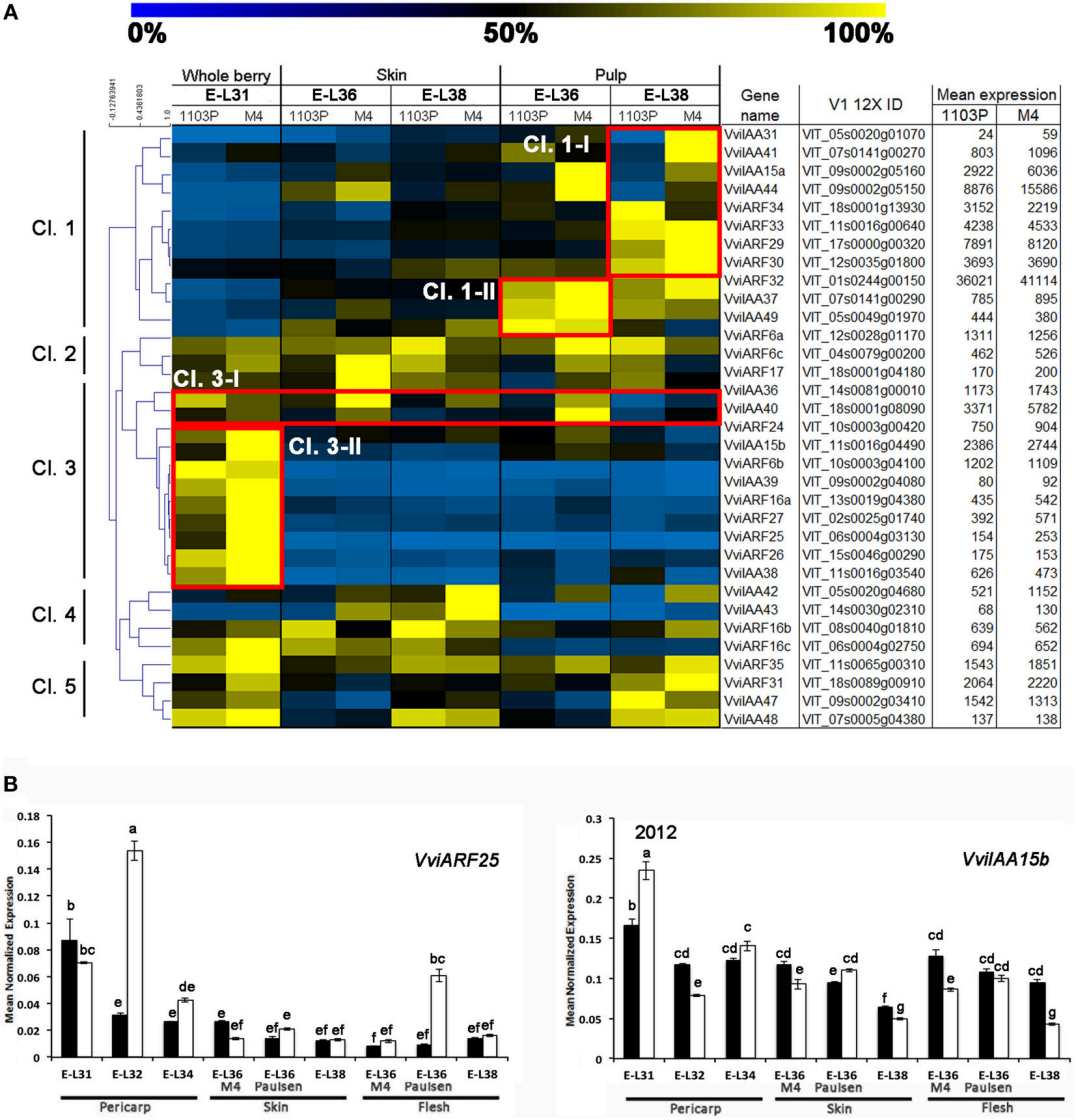
**(A)** Heat-map showing the expression of auxin-related *VvARF* and *VvAux/IAA* genes based mRNA-Seq data. The expression values were calculated as a percentage related to the developmental stage showing the highest expression value (100 and 0% for yellow and blue colors, respectively) within all mRNA-seq datasets considered (E-L31, E-L36, and E-L38). Proposed gene name (Supplementary Results S1), PN40024 V1 12X identifier, and mean of normalized counts are also reported. **(B)** quantitative RT-PCR analyses on two genes belonging to Cl.3-II (*VviARF25* and *VviIAA15b*) in flesh and skin of berries sampled from both 1103P/CS (solid bars) and M4/CS (empty bars) graft combination in 2012 growing season Results are shown as means and SE for three biological replicates. Bars indicate SE. Different letters indicate statistically significant differences (*P* = 0.05) by Duncan's new multiple range test.

Based on the notion that Aux/IAA and ARF TFs interact with each other to finely regulate the auxin- signaling pathway, we considered genes belonging to these families together. Figure 4A shows the expression and hierarchical clustering of a subgroup of *Aux/IAA* and *ARF* members, excluding those genes scarcely represented by mRNA-Seq reads, in order to avoid misinterpretation of results due to their contribution. Based on their expression profile, genes were divided into five clusters. The majority of the *Aux/IAA* and *ARF* genes were found in Cl.1 and 3. Most of the genes belonging to Cl.3 cluster, and specifically those found in the Cl3-II subgroup, were expressed exclusively at pre-véraison stage. This included both *Aux/IAAs* (*VviIAA15b*, −*38*, and −*39*) and *ARFs* (*VviARF6*, −*6b, 16, 24*, −*25*, −*26, and 27*) members. Although these genes showed a similar behavior in both genotypes, the induction observed in berries collected from CS/M4 was markedly higher than that observed in berries collected from CS/1103P. That was particularly true for *VviIAA15b, VviARF16b*. *VviARF25* and *VviARF27*. Only *VviIAA36* and *VviIAA40*, although belonging to Cl.3-I, were also expressed at E-L36, both in skin and pulp tissues. Similar to what was observed for members belonging to cluster Cl.3-II, the induction observed in CS/M4 berries was much higher compared to what observed in CS/1103P. The fact that Aux/IAA and ARF are known to physically interact to regulate the auxin signaling pathway and that *Aux/IAA* and *ARF* genes belonging to the Cl.3 cluster were strictly correlated in term of expression raises some questions about the possible interactions amongst them.

The opposite pattern was observed for members belonging to cluster Cl.1, mainly expressed in flesh and at those developmental stages following véraison. Genes belonging to cluster Cl.1-I were induced exclusively in the pulp of berries at ripening phase (apart from *VviIAA15a and* −*44* which were induced only in the pulp of CS/M4 berries at E-L36), whereas genes belonging to cluster Cl.1-II were induced at E-L36 and E-L38.

Less clear was the behavior of genes belonging to clusters Cl.2, Cl.4, and Cl.5, although the latter appeared to be composed of genes preferentially expressed at pre-véraison and ripening stages in pulp. Biochemical and colorimetric data showed that the differences in the rate of berry development between the two graft combinations were limited to the onset of ripening. For this reason we focused our attention on those *Aux/IAA* and *ARF* genes belonging to cluster Cl.3-II, characterized by a higher expression at pre-véraison stages and whose differential behavior could be associated to the different ripening rate observed in the two graft combinations. In order to validate and expand results obtained from the mRNA-Seq data, we performed a quantitative RT-PCRs on *VviIAA15b* and *VviARF25*, representing those Cl. 3-II members showing the highest difference in fold change between CS/1103P and CS/M4, at E-L31, 32, 34, 36, and 38. Both genes were showing the highest expression in CS/M4 at the pre-véraison stages (E-L31 and E-L32) (Supplementary Figure S3). In 2012, the expression profile of *VviARF25* and *VviIAA15b* was confirmed. This result reinforces the hypothesis for a role in the transition from the immature to mature fruit development stage.

### CS/1103P and CS/M4 berries show a shift in auxin homeostasis during ripening

**Figure 5:**
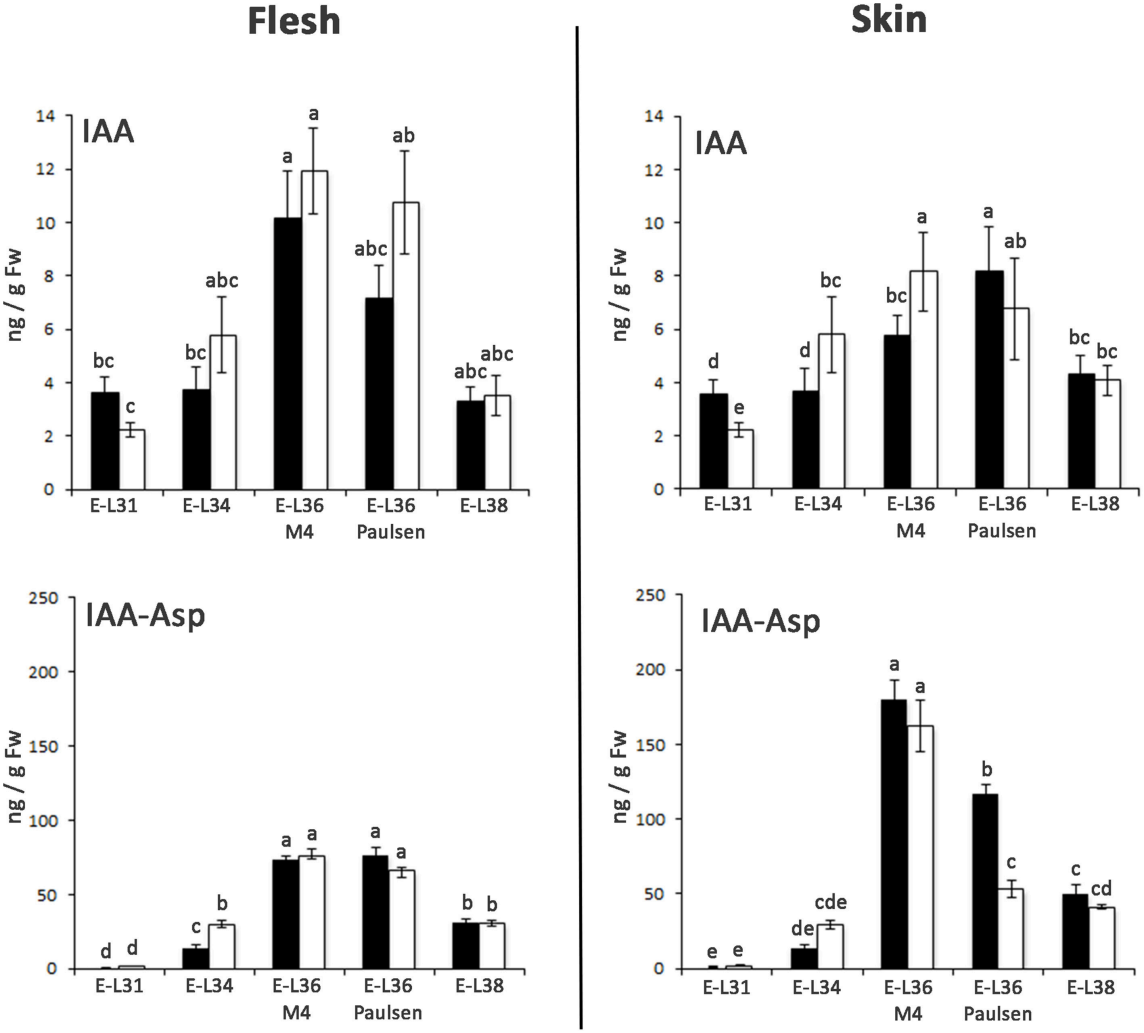
**Changes in levels of free IAA and IAA-Asp in flesh and skin of berries sampled from both 1103P/CS (solid bars) and M4/CS (empty bars) graft combination in 2011**. IAA and the IAA-Asp were quantified by mean of LC-MS/MS at E-L31, E-L34, E-L36 M4, E-L36 Paulsen, and E-L38 developmental stages. Bars indicate SE of four replicates. Mean followed by the same letters are not statistically different at *P* = 0.05 (Duncan's multiple-range test).

The positive relationship between auxin level and *Aux/IAA* transcription has been well documented (Zenser et al., 2001). Based on this observation, to investigate whether the differences observed in the expression of *ARF* and *Aux/IAA* genes in CS/M4 and CS/1103P berries were associated to differences in auxin homeostasis, we measured the level of free and conjugated IAA in berry samples collected in 2011. The level of free (IAA) and conjugated (IAA-Asp) auxin is shown in Figure 5. In berry flesh, no significant differences in IAA and IAA-Asp content were found between the two graft combinations. In comparison, at the skin level, their accumulation appeared to follow different kinetics. Indeed, M4 induced a significantly higher accumulation of free auxin at 65 (E-L34) and 72 (E-L36 M4) DABF, compared to that detected in CS/1130P berries. Later on, the two graft combinations showed similar level of IAA. As for IAA-Asp, CS/M4 showed a quantity two-fold higher than 1103P at E-L34 stage. During véraison CS/1103P berries appeared to accumulate more IAA-Asp than CS/M4 while at harvest no significant differences were observed.

**Figure 6:**
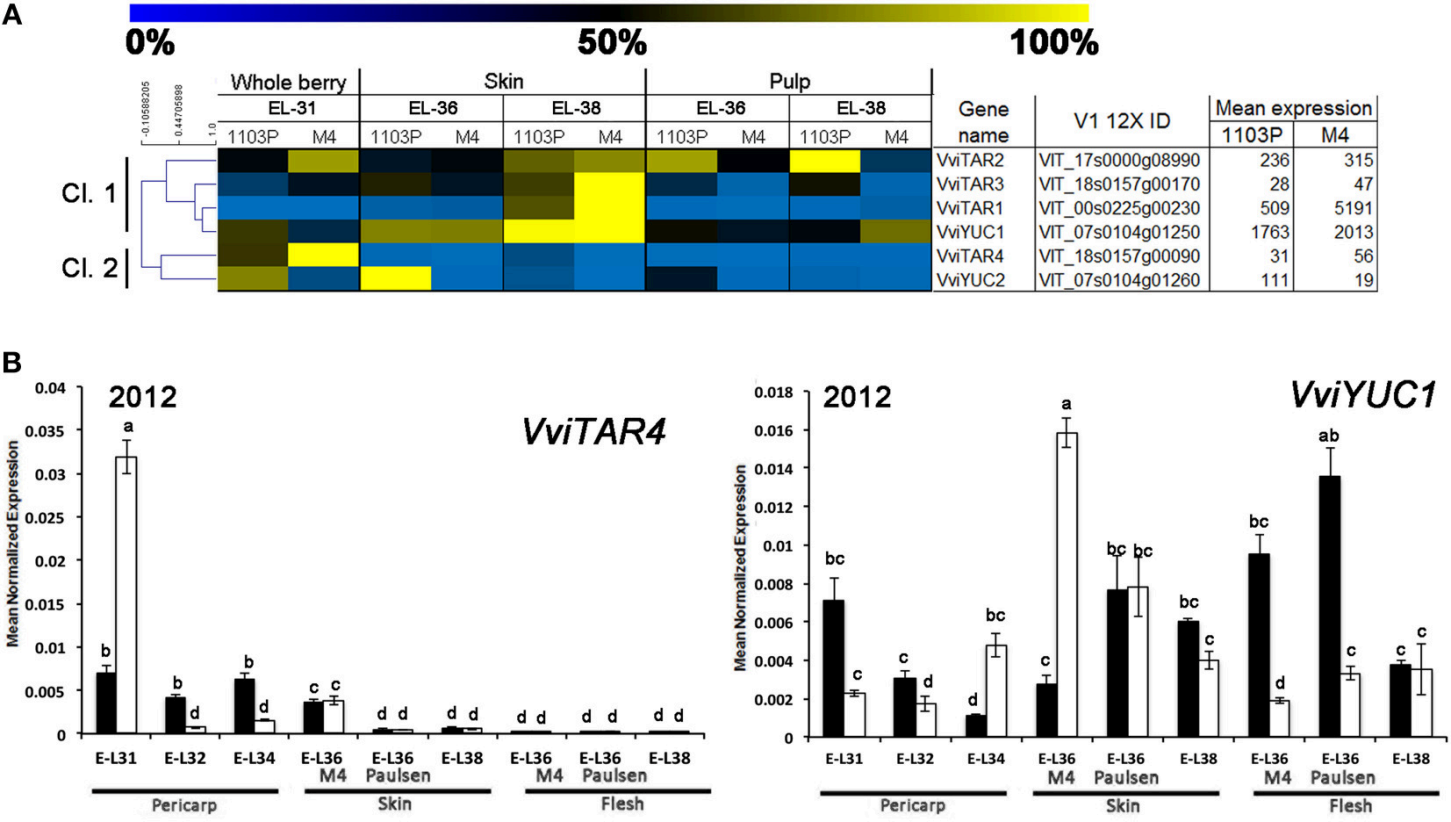
**(A)** Heat-map showing the expression of auxin-biosynthetic genes based mRNA-Seq data. The expression values were calculated as a percentage related to the developmental stage showing the highest expression value (100 and 0% for yellow and blue colors, respectively) within all mRNA-seq datasets considered (E-L31, E-L36, and E-L38). Proposed gene name (Supplementary Results S1), PN40024 V1 12X identifier, and mean of normalized counts are also reported. **(B)** Quantitative RT-PCR analyses on *VviTAR4* and *VviYUC1* in flesh and skin of berries sampled from both 1103P/CS (solid bars) and M4/CS (empty bars) graft combination in 2012 growing season. Results are shown as means and SE for three biological replicates. Bars indicate SE. Different letters indicate statistically significant differences (*P* = 0.05) by Duncan's new multiple range test.

**Figure 7:**
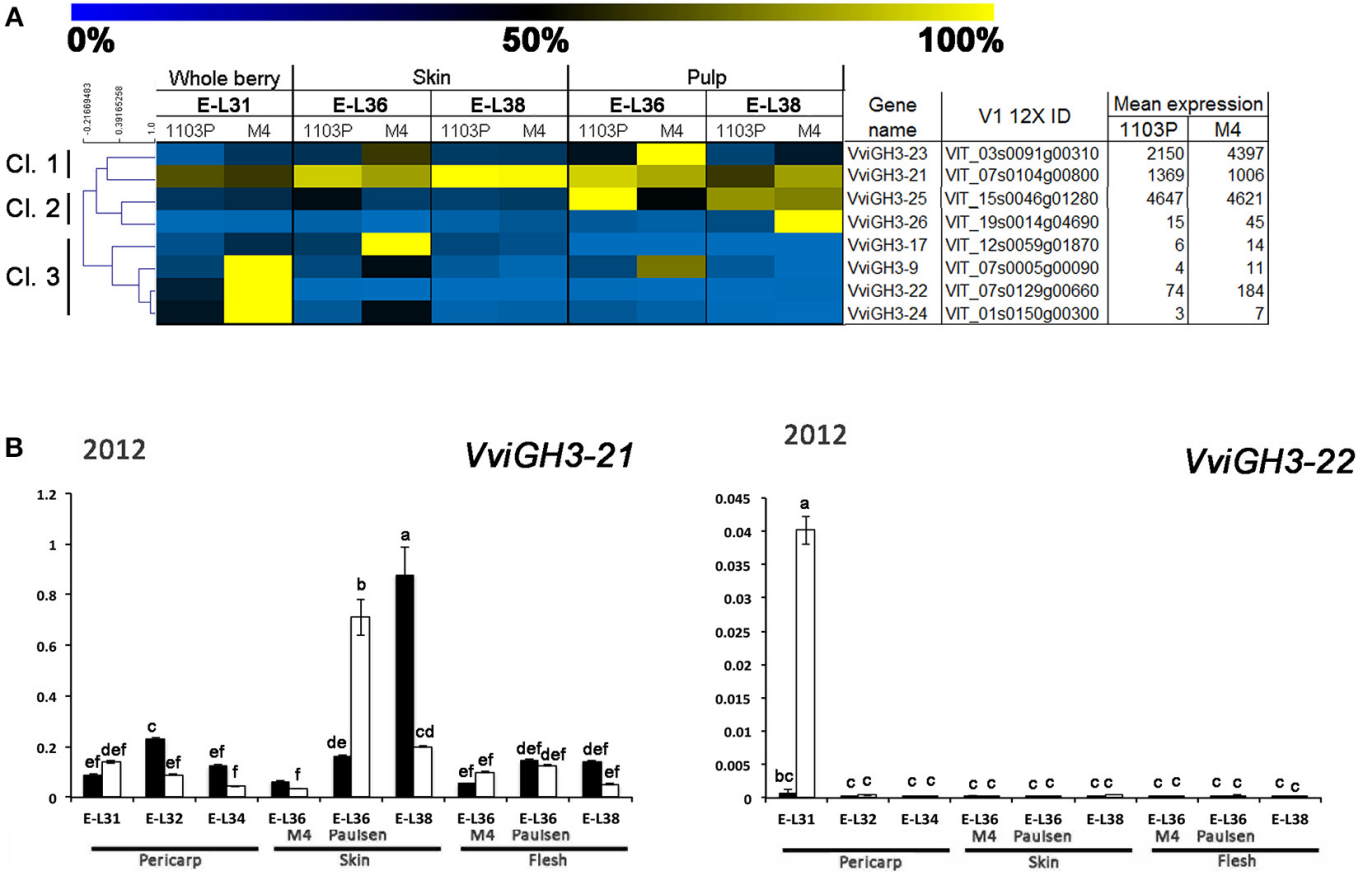
**(A)** Heat-map showing the expression of *VviGH3* genes based mRNA-Seq data. The expression values were calculated as a percentage related to the developmental stage showing the highest expression value (100 and 0% for yellow and blue colors, respectively) within all mRNA-seq datasets considered (E-L31, E-L36, and E-L38). Proposed gene name (Supplementary Results S1), PN40024 V1 12X identifier, and mean of normalized counts are also reported. **(B)** Quantitative RT-PCR analyses on *VviGH3-9* and *VviGH3-22* in flesh and skin of berries sampled from both 1103P/CS (solid bars) and M4/CS (empty bars) graft combination in 2012 growing season. Results are shown as means and SE for three biological replicates. Bars indicate SE. Different letters indicate statistically significant differences (*P* = 0.05) by Duncan's new multiple range test.

The relative mRNA-Seq expression of genes involved in auxin biosynthesis and conjugation is graphically represented in Figures 6A,7A, regardless whether they were or not included amongst those DEGs obtained by the multifactorial analysis. Considering genes involved in auxin biosynthesis, the hierarchical clustering on mRNA-Seq data split auxin-biosynthetic genes into two sub-groups (Figure 6A). In this regard, considered genes can be divided into early-expressed (E-L31, Cl.2) in whole berries and late-expressed (E-L36 and E-L38, Cl.1) in skin. Considering their high mRNA-Seq expression (Figure 6A), *VviYUC1* (Cl. 1) and *VviTAR4* (Cl. 2) were selected for qRT-PCRs (Figure 6B). Both in 2011 (Supplementary Figure S3) and 2012, (Figure 6B), the expression profile of *VviTAR-4* and *VviYUC1* genes was assessed. *VviTAR-4* was found to be more highly expressed at pre-véraison stages in CS/M4 than in CS/1103P, while *VviYUC1* transcripts were more highly accumulated at E-L36 in CS/M4 and at E-L38 in CS/1103P.

A closer relationship was observed between IAA-Asp and *GH3* transcript levels (Figures 5,7A,B). Hierarchical cluster analysis (Figure 7A) led to the identification of three main subgroups. Amongst them, Cl. 3 represented the most interesting one, being characterized by the presence of genes such as *VviGH3*-9, *VviGH3-24*, and *VviGH3-22*, which were strongly expressed at pre-véraison stage in CS/M4 but not in CS/1103P. The behavior of one of these genes (*VviGH3-22*) was also confirmed by qPCR in 2011 (Supplementary Figure S3) and 2012 (Figure 7B). Less obvious was the expression of genes belonging to other clusters. We also considered the expression pattern of *VviGH3-21*, which, based on mRNA-Seq data (Figure 7A) appeared to be highly expressed in skin tissue of CS/1103P at E-L38. Quantitative RT-PCR validated this observation (Supplementary Figure S3 and Figure 7B).

### The expression profile of flavonoid-related genes parallels the levels of IAA-Asp in grape skin

**Figure 8:**
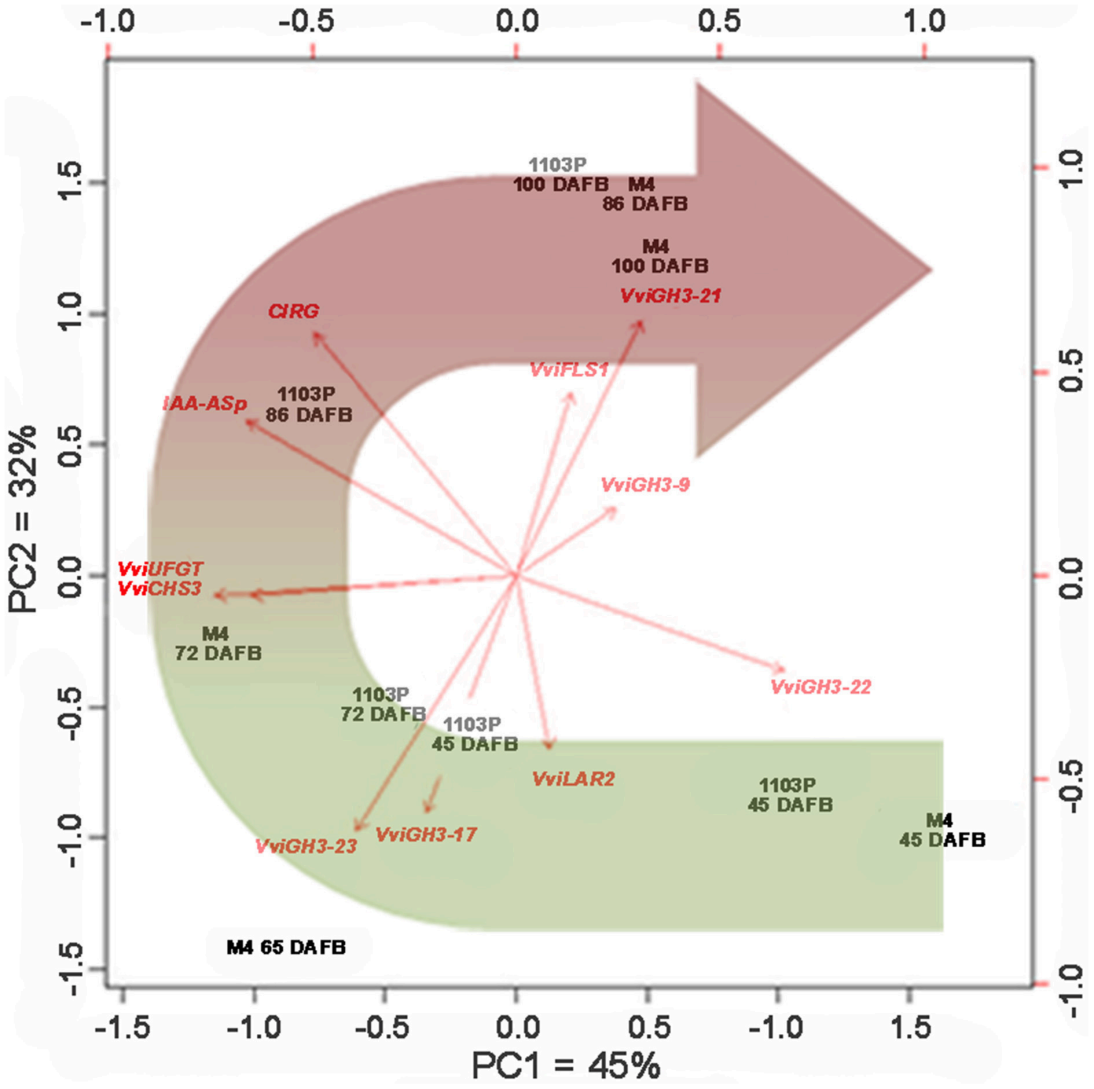
**CS/M4 and CS/1103P samples distribution according to a PCA analysis carried out on *VvGH3s* and flavonoid-related qRT-PCR data, and colorimetric parameters (CIRG index)**. Percent of variance for each component on the corresponding axes and effect of considered parameters on samples distribution (red arrows) are also showed

CS/1103P and CS/M4 clearly display a differential regulation of auxin metabolism, as showed by free and conjugated IAA quantification (Figure 5) and molecular analyses (Figures 4, 6, 7). This different behavior could lead to a different rate in the berry development and ripening (Figure 1) particularly evident in the skin, as pointed out by colorimetric measurements (Figure 1C, Supplementary Figures S1, S2B). In fact, skin color evolution and CIRG index indicated a delay in CS/1103P skin pigmentation and accumulation of flavonoids in comparison to what was observed in CS/M4 berries. The change in skin pigmentation was paralleled by changes in the transcript accumulation of flavonoid biosynthesis (phenylalanine ammonia lyase, *VviPAL3-like;* chalcone synthase *3, VviCHS3*; flavanol synthase *1, VviFLS1*; leucoanthocyanidin reductase 1 and 2, *VviLAR1* and *VviLAR2*), flavone- and flavonol- (*VviUFGT*) related genes (Supplementary Figure S4). In particular, the expression of *VviPAL3-like, VviCHS3, VviLAR2*, and *VviUFGT* occurred earlier (E-L36 M4) and was higher in CS/M4 berries than in CS/1103P ones. To associate changes in IAA-asp concentration, CIRG value and *GH3* and flavonol-related gene expression to the evolution of skin pigmentation in CS/M4 and CS/1103P berries during ripening, a PCA analysis was carried out on samples collected at pre-véraison (E-L31 and E-L34 corresponding to 45 DAFB and 65 DAFB), during véraison (E-L36, 72 DAFB for M4 and 86 DAFB for 1103P), and ripening (E-L38, 100 DAFB; Figure 8). The first two PCA components explained the 77% of the variance, contributing with similar weights (PC1 = 45% and PC2 = 32%). Examination of the scores and loadings plots for PC1 vs. PC2 showed that the distribution of samples was based on the fruit developmental stages. Samples collected at the pre-véraison stage were clearly separated from those collected during véraison and ripening phases. The PCA analysis also revealed that the early pre-véraison stage (45 DAFB) was strictly associated to the accumulation of *VviGH3-22* transcripts, whereas the induction of other genes, such as *VviLAR2, VviGH3-23*, and *VviGH3-17*, marked the late pre-véraison stage (68 DAFB) in both graft combinations. At 72 DAFB, by the time CS/M4 berries almost completed the change of skin color (accompanied by the induction of *VviCHS3* and *VviUFGT* transcription), CS/1103P was still in pre-véraison stage and reached mid/late véraison (marked by the accumulation of IAA-asp and flavonoids) at 86 DAFB. However, despite the delay displayed in ripening rate, CS/1103P berry collected at 100 DAFB clustered, on the basis of skin parameters, with those of CS/M4 suggesting their recovery of ripening progression. (Figure 7B).

## Discussion

The present study evaluated the effect of two grapevine rootstocks, the commonly used and vigorous 1103 P (*V. berlandieri* × *V. rupestris*) and the experimental genotype M4 [(*V. vinifera* × *V. berlandieri*) × *V. berlandieri* cv. Resseguier n. 1], on *V. vinifera* cv. Cabernet Sauvignon berry development and ripening. The M4 genotype, developed by the DISAA department (University of Milan) was selected for its high tolerance to water deficit and salt exposure and was classified as a medium-vigorous rootstock (Meggio et al., 2014; Corso et al., 2015). The aim of the present study was to shed light on the impact of rootstocks on the scion berry development from a physiological and molecular point of view.

In our study we showed that the rate of berries ripening in CS plants grafted onto M4 is faster (in terms of sugar accumulation and change of skin color) than that observed in the CS/1103P combination (Figure 1). These results are in agreement and partially explained by previous studies showing that the use of the high vigor rootstock 1103P is associated to an extension of the vegetative cycle and a delay in ripening (Koundouras et al., 2008; Gambetta et al., 2012). Biochemical (Figure 1B) and colorimetric data (Figure 1C, Supplementary Figure S1) were also supported by molecular ones (Figure 2). Multifactorial analyses conducted on mRNA-Seq data obtained from CS/1103P and CS/M4 berries at pre-véraison (E-L31), mid-late véraison (E-L36) and ripening (E-L38) indicated that the differential expression of 2358 genes (DEGs) is mainly affected by the rootstock (Figure 3; Supplementary Table S3). Enrichment analyses (Figure 3; Supplementary Table S4) evidenced that, amongst DEGs whose expression is influenced by the rootstock factor (R), many are associated to auxin-related functional categories. Amongst these categories the most prominent regarded genes involved in the auxin signal transduction. Auxin signal transduction is mediated by *Aux/IAA* and *ARF* genes (Pierre-Jerome et al., 2013), which appeared both differently modulated in the two graft combinations (Figure 4). In particular, those *Aux/IAA* and *ARF* genes more expressed at pre-véraison stage showed a higher accumulation of their transcripts in CS/M4 (Figure 5). Amongst these was *VviARF25*, very similar to the response repressor AtARF4 (Supplementary Results S1), which was recently demonstrated to interact with almost all Aux/IAAs and to show broad co-expression relationships with *Aux/IAA* genes (Piya et al., 2014). However, Kepinski (2007) suggested that specific pairs of AUX/IAAs and ARFs function depending on the tissue and developmental stage considered. In our study *VviARF25* was co-expressed with *VvIAA15b*, −*38*, and −*39* (Figure 5). Thus, they products could interact forming putative pairs able to control the expression of auxin-inducible genes at the pre-véraison stage. This result suggests that the rootstock-dependent modulation of auxin action could be involved in the control of berry development rate, similarly to what already hypothesized by Cookson and Ollat (2013) for what concerns the shoot development. At this regard, it was observed that many genes belonging to the functional categories IAA/auxin were both up- and down-regulated in shoot apical meristems of CS grafted on two different rootstocks, respectively Riparia Gloire de Montpellier and 1103P.

Together with genes involved in auxin action, CS berries grafted on 1103P and M4 rootstocks also showed different patterns of induction for genes involved in auxin biosynthesis and conjugation. Regarding auxin biosynthetic genes, *VviTARs* and *VviYUCCAs* were expressed at pre-véraison stages and during véraison, as previously observed by Böttcher et al. (2013). Although showing expression patterns only partially overlapping in the two growing season (Figure 6) considered, it's clear that the two rootstocks determined a different modulation of their transcript levels. Expression of auxin biosynthetic genes is partially overlapped with the differential IAA accumulation observed between CS/M4 and CS/1103P. This is particularly true for skin tissue, where M4 induces a significantly higher accumulation of auxin at 65 (E-L34) and 72 (E-L36 M4) DABF, compared to CS/110P (Figure 5). It is worth to note that difference in ripening rate between the two graft combinations parallels IAA-Asp accumulation in the skin and was coupled to an earlier and higher expression of genes involved in auxin biosynthesis (e.g., TAR4; Figure 6B) and action (i.e., *VviARF6a, VviARF6c, VivARF16a*, and *VviARF34*, Figure 4A) in CS/M4 berries. Together with auxin biosynthesis, the conjugation process represented an important auxin homeostatic mechanism at pre-véraison stage. At this regard, of particular interest was the behavior of genes involved in auxin conjugation (*VvGH3s*), especially for those ones showing a peak of expression in pre-véraison phase (E-L31). Amongst these, *VviGH3-22* (VIT_07s0129g200660) was specifically expressed in pre-véraison stage in both graft combination (Figure 7) and at very low levels in all other developmental stages. This gene corresponds to *GH3*-2 in the nomenclature proposed by Böttcher et al. (2011) (Supplementary Results S2), which described a similar behavior in CS and claimed it to be the most responsible gene for auxin homeostasis in pre-véraison. Both mRNA-Seq and qPCR analyses pointed out that *VviGH3-22* transcript is differentially accumulated between the two graft combinations, being much more expressed in CS/M4 than CS/1103P. This observation is in agreement with the higher ability of CS/M4 berries to conjugate IAA at pre-véraison stages (E-L34, Figure 5). Considering that the IAA-Asp conjugate might also represent a ripening signal in grapes (Böttcher et al., 2013), the early accumulation observed in CS/M4 at E-L34 (Figure 5) could be associated to the earlier onset of ripening in this graft combination. This shift in IAA-Asp accumulation was maintained along the whole ripening although was evident only at the skin level, where a higher accumulation of IAA-Asp was observed in CS/1103P. The different kinetic of IAA-Asp accumulation at these late stages could be associated to the expression pattern of another *GH3* gene, namely *VviGH3-21*. This gene, corresponding to *GH3-8* described in Böttcher et al. (2011), encodes for a deduced protein representing an out layer respect to the other GH3 members identified in grapevine and up to now its expression has not been investigated. In the present study *VviGH3-2*1 was found to be mainly expressed in the skin (Figure 7B) and at later stages compared to other *GH3* members. This observation could be associated to the later IAA conjugation observed in the skin and would be consistent with a delay in the ripening programme progression of skin respect to pulp as previously reported (Castellarin et al., 2011; Lijavetzky et al., 2012). The high expression of *VviGH3-21* in late ripening phases of CS/1103P berries compared to what observed in CS/M4 ones could be the result of the rootstock ability to modulate the transcriptome of grape berry. Recently, it was reported that the rootstock is able modulate the expression of a number of genes in the scion (Cookson and Ollat, 2013; Berdeja et al., 2015; Kumari et al., 2015). In particular, Berdeja et al., (2015) reported that, in berries of Pinot noir plants undergoing water stress condition, the transcript level of genes involved in jasmonate metabolism changes based on the rootstock utilized (Kober 125 AA or Ritcher 110). However, our results pointed out that skin colorimetric parameters of ripe berries (E-L 38) are similar between the two graft combinations suggesting an acceleration of ripening induced by 1103P rootstock at last stages of maturation. This result, although obtained in different graft combinations, could be associated to the observations reported by Gouthu et al. (2014) which showed that in a cluster, during the last phase of fruit developmental cycle, the ripening rate of under-ripe berries is higher than that measured in the ripest berries to reach a synchronized development. This result indicated that, although starting with different timings, the ripening transcriptional programme has to be completed in a genetically defined temporal window independently by exogenous factors affecting the early phases of berry ripening initiation. Similarly, our results pointed out that rootstock is able to modulate the ripening rate but, later on, the genetic control of berry ripening is the main driving force leading to the achievement of full maturity. This result reinforces the assumption that the plasticity of ripening-related genes is mainly modulated by the developmental phase and almost unaffected by external stimuli (e.g., environmental conditions; Dal Santo et al., 2013). Nevertheless, the ripening initiation signal is not only linked to hormone dynamic but also to the status of sugar content, which in turn depends on the competition between the different sinks (Ho, 1988; Bobeica et al., 2015). In the sense using rootstocks characterized by different vigor could determine temporal variation in the duration of ripening programme influencing the relations between fruit and shoot sinks (favoring the shoot development in the case of vigorous rootstock) and, as consequence, the sugar uptake toward them.

## Conclusions

Data presented here suggest that the regulation of auxin level is differently affected in the two scion /rootstock combinations and this is positively correlated with a different rate of grape berry development and ripening. The identification of links between signals controlling berry ripening and rootstock would be of great importance for a better understanding of the influence of rootstock on the scion performance. It has been postulated the ability of rootstock to induce high auxin levels in scion buds as the factor inducing the positive effect of vigorous peach rootstocks on scion branching (Sorce et al., 2006). Nowadays it is becoming evident that throughout the graft union occur a dynamic exchange of mobile signals [transcription factors, mRNAs, regulatory micro RNAs (miRNAs), small interfering RNAs (siRNAs), peptides, and proteins] between scion and rootstock (Haroldsen et al., 2012). Among mobile signals, small non-coding RNAs could play an important role in the regulation of complex processes as fruit development and ripening because of their ability to regulate gene expression in a much more tuneable manner (Vazquez et al., 2010). In this context, there are many evidences that the use of small RNAs, aside from pathogen resistance, down–regulation, and/or epigenetic modification of transcripts and genetic networks, could influence scion-specific characteristics, such as flowering time and fruit production or quality (Haroldsen et al., 2012). In addition to hormones (data presented here) investigations on the role of small RNAs, as well as, those of other signal molecules could help to better clarify the impact of rootstock on berry scion development and ripening.

## Author contributions

MC, AV, ML, and CB developed the concept of the paper, wrote the paper, and together with MZ, EM, NV, MB, and GV performed the whole transcriptome and bioinformatic analyses; TN and FZ carried out qRT-PCR analyses, MM and SM performed auxin quantification and FM collected and analyzed meteorological data. All authors discussed and commented on the manuscript.

## Funding

This study was supported by the AGER “SERRES” project, grant n° 2010–2105. The cooperation among the international partners was supported by COST Action FA1106, Quality fruit.

### Conflict of interest statement

The authors declare that the research was conducted in the absence of any commercial or financial relationships that could be construed as a potential conflict of interest.

## Supplementary material

Supplementary Data S1 **Meteorological data originate from the Regional Agency for the Environmental Protection of Veneto (ARPAV), Italy**. Climatological data of decadal temperatures, rainfall, and temperature excursions for 2011–2012 compared to the period 1992–2012 are reported.

Supplementary Figure S1 **Colorimetric results of CS/1103P and CS/M4 berry**. Analyses were carried out at four time points corresponding to 45, 72, 86, and 100 DAFB.

Supplementary Figure S2 **Samplings of berries grown in both 1103P/CS and M4/CS graft combinations were performed at different stages of berry development and ripening**. Growing season: 2012. **(A)** Soluble solids content in CS/M4 (squares) and CS/1103P (circles) throughout fruit development. **(B)** CIRG values of CS/M4 (square) and CS/1103P (circle) graft combinations throughout fruit development. Bars represent the SD of 100 berries. CS/M4 and CS/1103P data from samples collected at the same DAFB were statistically treated using Student's *t*-test (^*^*P* < 0.05; ^**^*P* < 0.01).

Supplementary Figure S3 **Quantitative RT-PCR analyses on the following *VviYUC1, VviTAR4, Vvi ARF25, VviIAA15b, VviGH3-21*, and *VviGH3-22* performed in flesh and skin of berries sampled from both 1103P/CS (solid bars) and M4/CS (empty bars) graft combination in 2011 growing season**. Results are shown as means and SE for two biological replicates. Bars indicate SE. Different letters indicate statistically significant differences (*P* = 0.05) by Duncan's new multiple range test.

Supplementary Figure S4 **Quantitative RT-PCR analyses on the following flavonoid-related genes: *VviPAL3*-like (VIT_13s0019g04460, A), *VviCHS3* (VIT_05s0136g00260, B), *VviLAR2* (VIT_17s0000g04150, C), *VviFLS1* (VIT_18s0001g03430, D), and *VviUFGT* (VIT_16s0039g02230, E)**. Transcript levels in CS/1103P (black) and CS/M4 (white) berries are shown as means of normalized expression ±SD.

Supplementary Results S1 **Nomenclature of genes belonging to the grapevine ARF, Aux/IAA, and GH3 multigenic families**.

Supplementary Table S1 **Primers used for qPCR analyses. V1 12X identifier (V1 12X ID), gene name, forward (FW sequence) and reverse (RV sequence) sequences are reported**.

Supplementary Table S2 **(A)** Sequencing and alignment statistics. Sample name (Sample), replicate, paired-end Tag, total number of produced reads (Total), filtered reads after trimming (Filtered), good-quality reads (# Good), aligned and percentage of aligned reads (% Aligned), number of alignment (# alignment) are reported. **(B)** Summary of read number after pairing (F3 + F5). Sample name (Sample), library name (LibName), number of unique reads after pairing (Single), percentage of sequences that aligned on a gene (% ReadsOnGene), number of gene with at least five reads (# of genes) and percentage of genes with at least five reads (% of genes).

Supplementary Table S3 **List of DEGs influenced by Rootstock (R), Tissue (T), and Phenological Phase (PP) components**. Numbers of DEG are given in brackets. PN40024 V1 12X annotation (V1 12X ID) and functional annotation (Funct. annot.) of DEGs are reported. Statistical analysis were carried out according to an FDR adjusted *p*-value lower than 0.05.

Supplementary Table S4 **Over-enriched GO terms of genes differentially expressed influenced by rootstock (R), tissue (T), and phenological phase (P) factors**. For each term, the GO identifier (GO-ID), the complete Gene Ontology term (Description), the *p*-value and the FDR-corrected *P*-value (corr *p*-value) of the Fisher's exact test, the numbers of sequences in the test set (x) are provided.

Supplementary Table S5 **Expression values (mean normalized mRNA-seq counts) of all 56 auxin-related genes**. Gene family, V1 12X annotation, gene name are reported. Graft combination (CS/1103P and CS/M4), considered tissue (whole berry, skin, and pulp) and phenological phase (E-L31, E-L36, and E-L38) are also indicated.
