# Supplementary material for: Grapevine Rootstocks Differentially Affect the Rate of Ripening and Modulate Auxin-Related Genes in Cabernet Sauvignon Berries
Source: Front Plant Sci. 2016 Feb 9;7:69. doi: 10.3389/fpls.2016.00069 (PMC4746306; doi:10.3389/fpls.2016.00069)
Supplement: Supplementary Data S1 — Meteorological data originate from the Regional Agency for the Environmental Protection of Veneto (ARPAV), Italy. Climatological data of decadal temperatures, rainfall, and temperature excursions for 2011–2012 compared to the period 1992–2012 are reported. [file SupplementaryDataS1.PDF]

**Supplementary Data S1. Meteorological data originate from the Regional Agency for the Environmental Protection of Veneto (ARPAV), Italy. Climatological data of decadal temperatures, rainfall and temperature excursions for 2011-2012 compared to the period 1992-2012 are reported.**

Time series data were processed and the daily total precipitations and minimum (Tmin), mean (Tavg) and maximum (Tmax) air temperatures were computed each year within the range 1992-2012. The decade climatology was computed as the average of all the values for the all the 15 decades (154 days) of grapevine vegetative cycle (May-September) for the data period of 1992–2012. The decade climatology and its variability are presented in Figure S1 and Figure S2. The decadal intra-seasonal anomalies for the seasons 2011 and 2012 of all the variables (Tmin, Tavg, Tmax, rainfall, temperature excursion) were computed by subtracting the daily climatology from the decadal actual value and results are reported in Figures A and B.
Significant inter-seasonal anomalies can be underlined among the two seasons 2011 and 2012 (Figure A). In particular, season 2012 was characterized by cold temperatures in the 2nd decade of May and thereafter by warmer temperatures compared to climatology in particular from the 2nd decade of June to the 22nd decade of July with air temperatures around 4 °C above the climatological average. Warmer temperatures around 3 °C above the climatological average were also registered in August. A quasi-specular behavior can be observed for season 2011, with warmer temperatures in early spring (3rd decade of May) and a significant drop of temperatures around 4 °C below climatological average in the 3rd decade of July. A further anomaly was registered in late summer 2012 with temperatures around 3-4 °C above climatological average from the 3rd decade of August to the end of the season.
Climatological anomalies for seasons 2011 and 2012 for rainfall are presented in Figure B. A similar behavior was observed among the two seasons with slight precipitation anomalies below the climatological average of around 20-25 mm throughout the season except for the 1st decade of June where in 2011 was registered a significant anomaly of around 110 mm above the climatological average compared to 2012. At the end of the season, cumulated anomalies of precipitations revealed quasi-specular behaviors among the two seasons with final values of -85 mm and -20 mm at the 3rd decade of September for 2011 and 2012 respectively.
Significant differences can be underlined among the two seasons on a temperature excursions basis. In particular seasons 2012 showed higher excursions above the climatological average compared to 2011. Greatest differences among the two seasons can be observed from the 1st decade of June to the 1st decade of August with higher temperatures excursions above the climatological average in 2012 compared to 2011 with lower excursions except for the 1st decade of June.

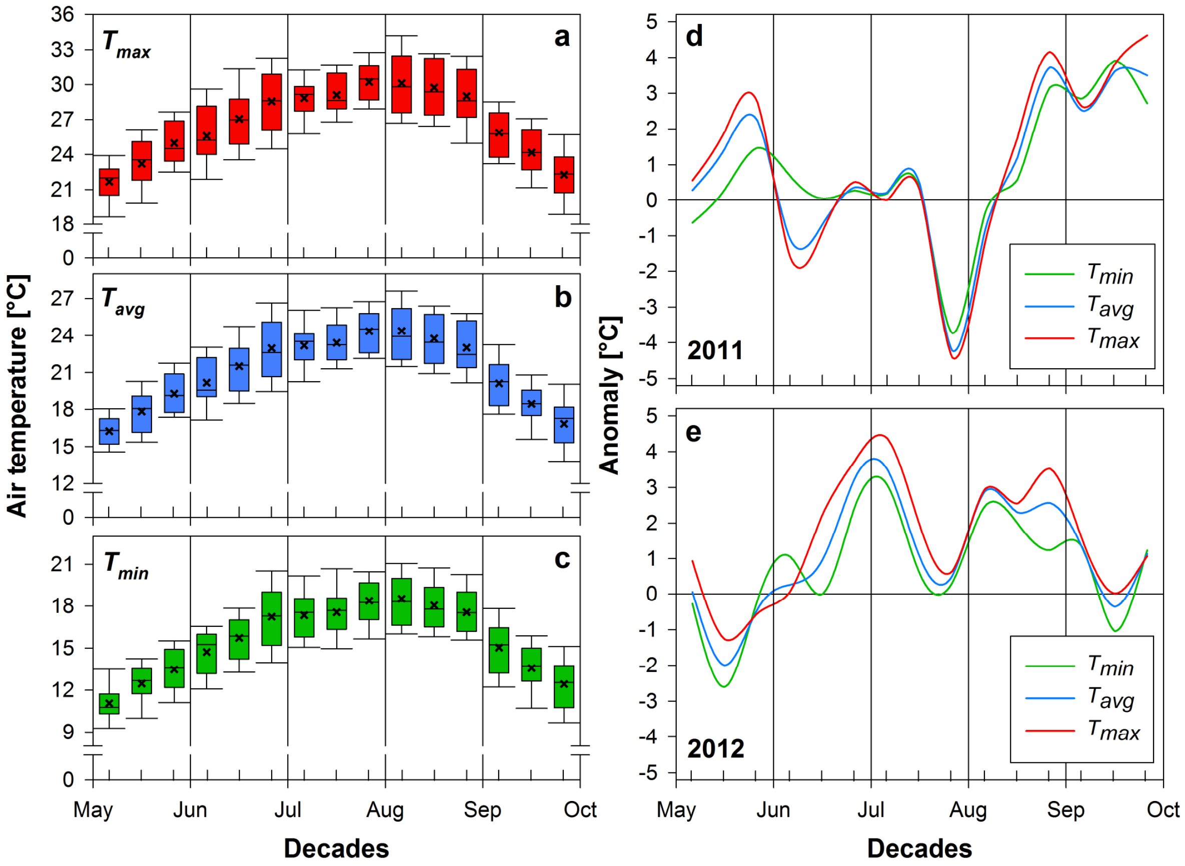


**Figure A.** Climatology of decadal temperatures: Tmin (a), Tavg (b) and Tmax (c) for the period 1992-2012 expressed with box and whiskers plot around median values (—), mean values (x). Climatological anomalies for Tmin, Tavg and Tmax for the seasons 2011 (d) and 2012 (e) on a decade basis.

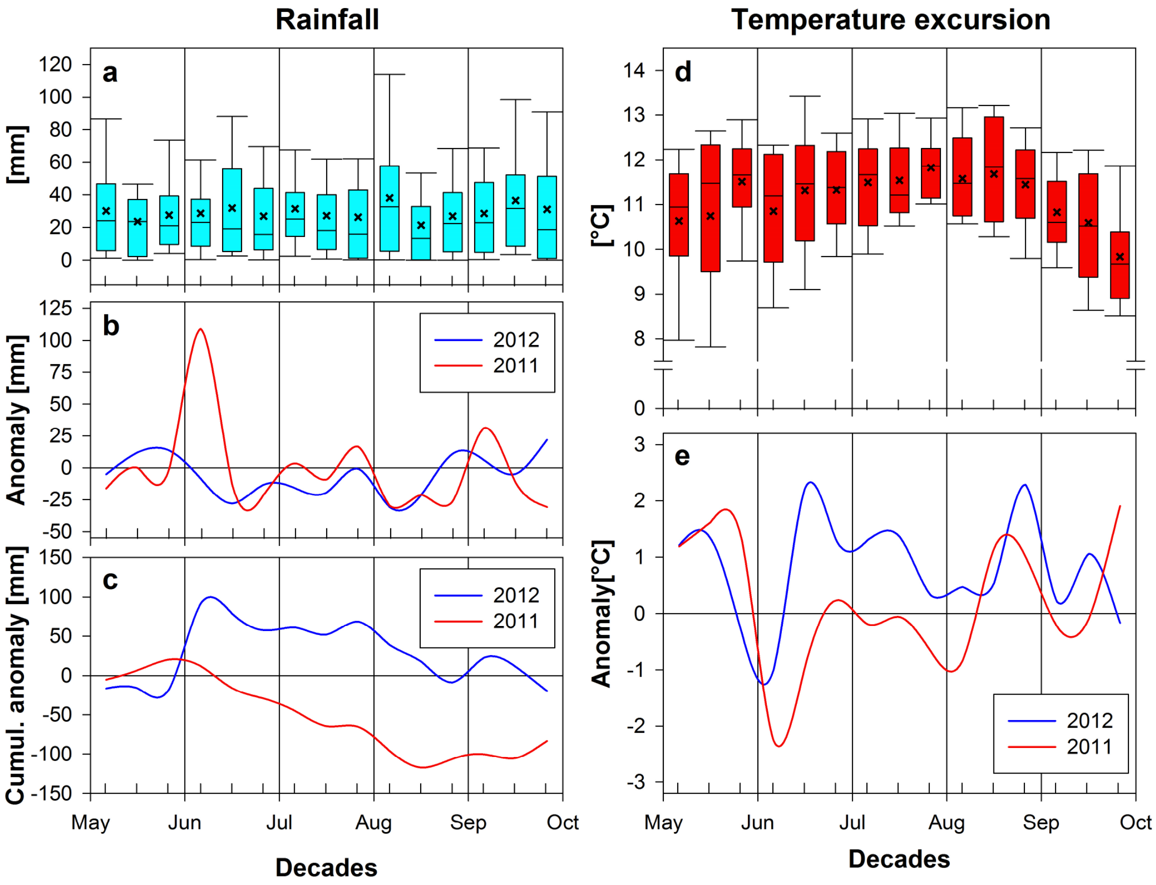


**Figure B.** Climatology of decadal rainfall (a) and temperature excursions (d) for the period 1992-2012 expressed with box and whiskers plot around median values (─), mean values (x). Climatological anomalies for rainfall (b), cumulated rainfall (c) and temperature excursions (e) for the seasons 2011 and 2012 on a decade basis.
