# Supplementary figures and images for: Grapevine Rootstocks Differentially Affect the Rate of Ripening and Modulate Auxin-Related Genes in Cabernet Sauvignon Berries
Source: Front Plant Sci. 2016 Feb 9;7:69. doi: 10.3389/fpls.2016.00069 (PMC4746306; doi:10.3389/fpls.2016.00069)

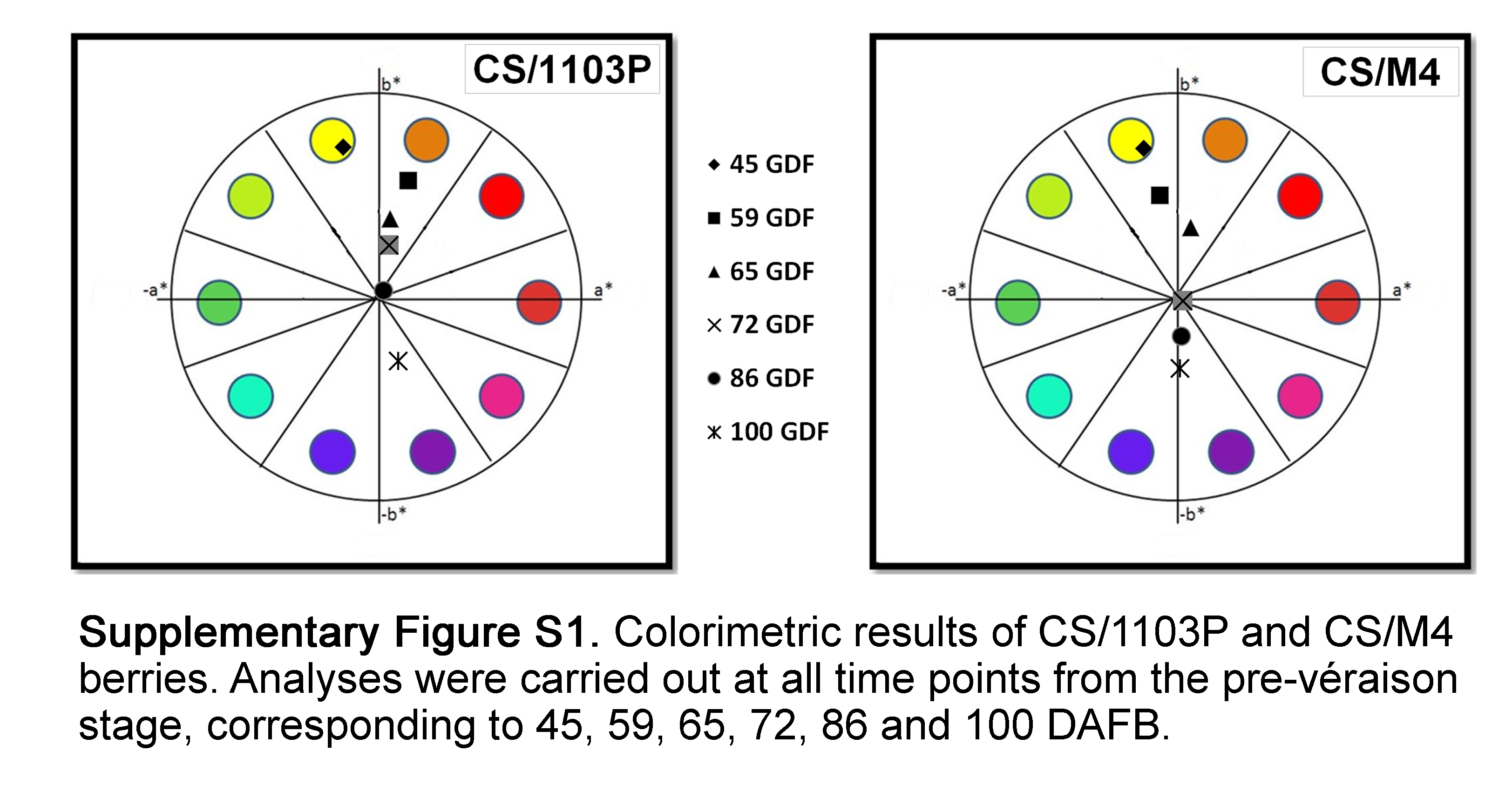

Supplement: Supplementary Figure S1 — Colorimetric results of CS/1103P and CS/M4 berry. Analyses were carried out at four time points corresponding to 45, 72, 86, and 100 DAFB. [file Image1.TIF]

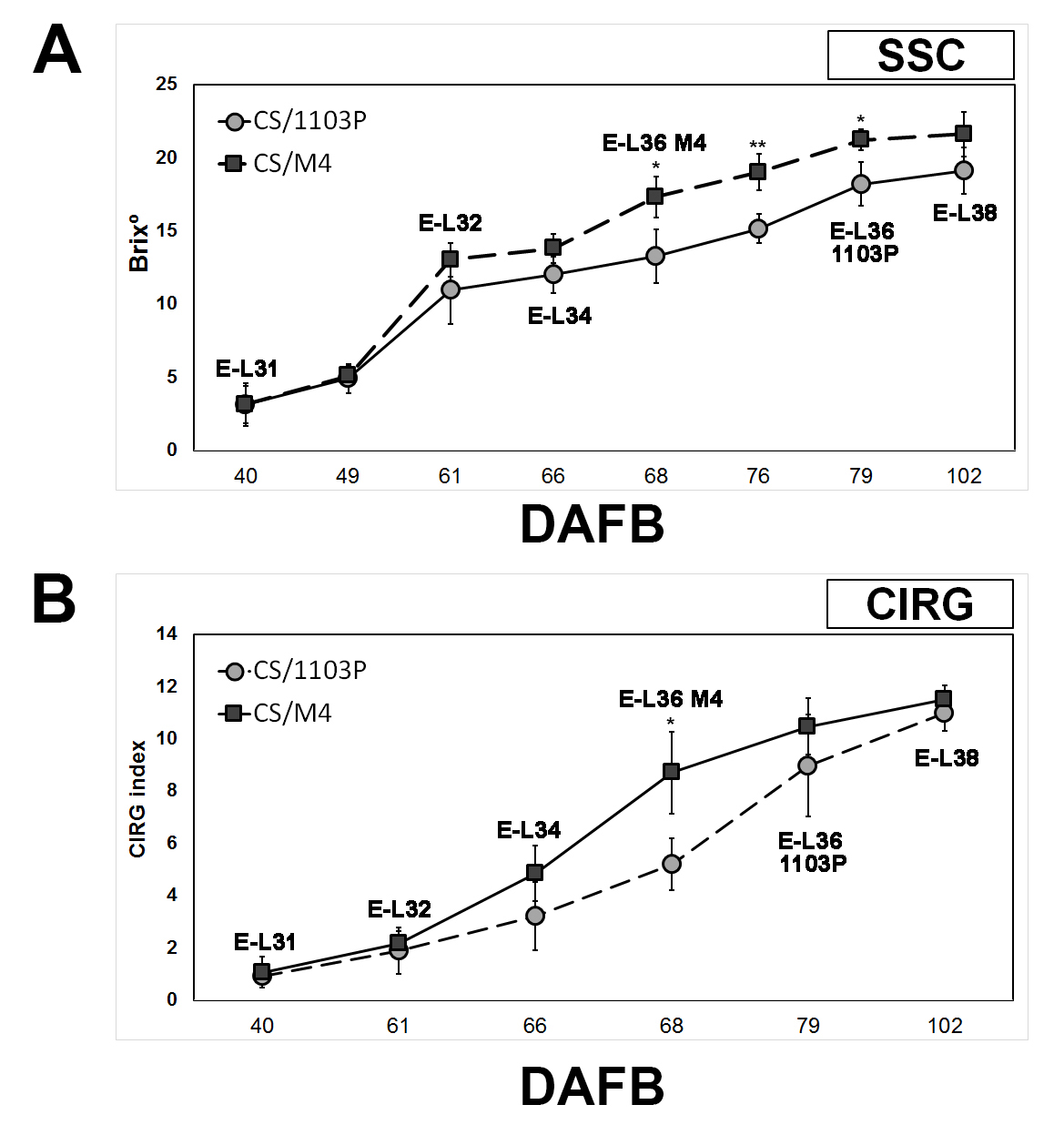

Supplement: Supplementary Figure S2 — Samplings of berries grown in both 1103P/CS and M4/CS graft combinations were performed at different stages of berry development and ripening. Growing season: 2012. (A) Soluble solids content in CS/M4 (squares) and CS/1103P (circles) throughout fruit development. (B) CIRG values of CS/M4 (square) and CS/1103P (circle) graft combinations throughout fruit development. Bars represent the SD of 100 berries. CS/M4 and CS/1103P data from samples collected at the same DAFB were statistically treated using Student's t-test (*P < 0.05; **P < 0.01). [file Image2.TIF]

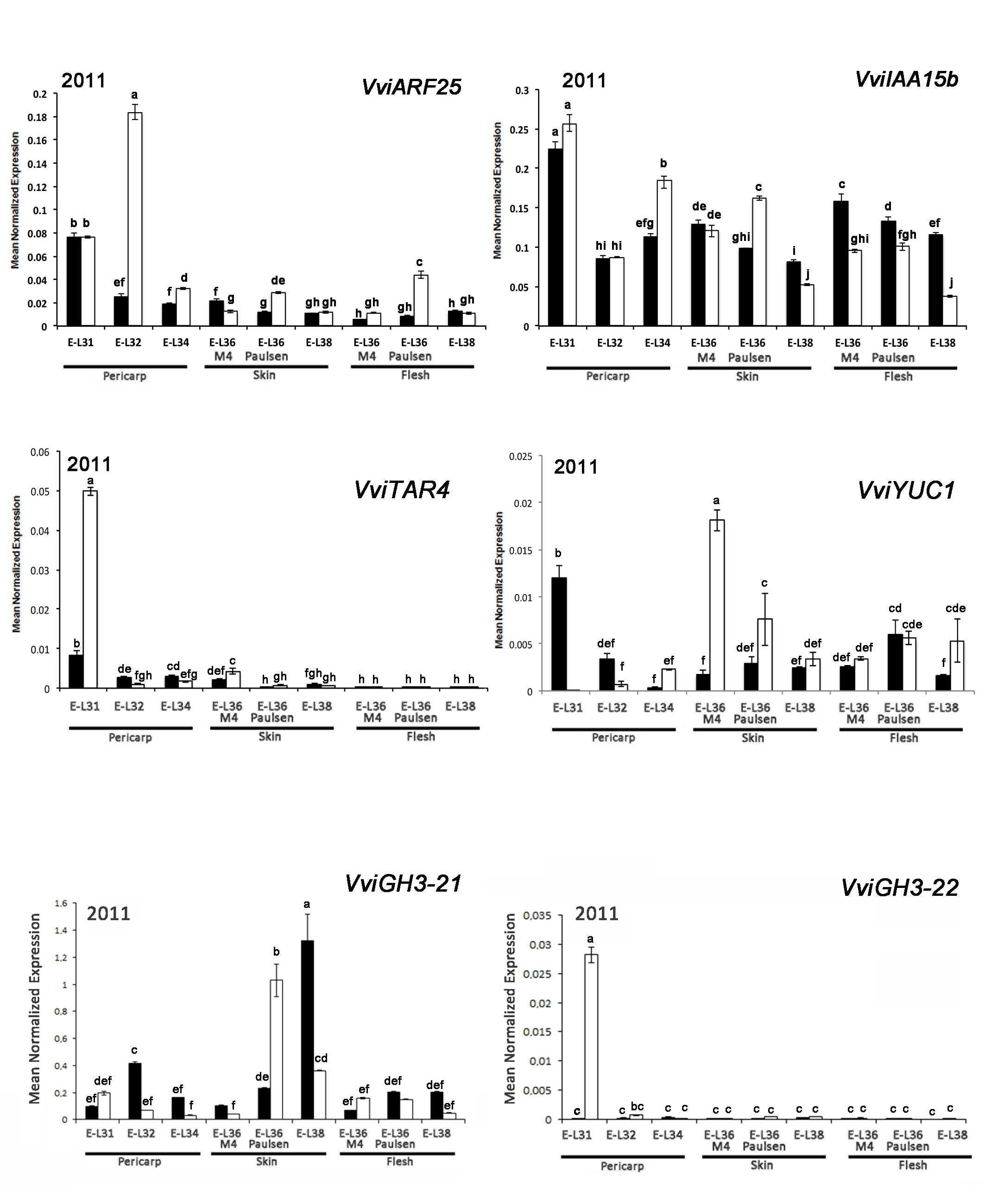

Supplement: Supplementary Figure S3 — Quantitative RT-PCR analyses on the following VviYUC1, VviTAR4, Vvi ARF25, VviIAA15b, VviGH3-21, and VviGH3-22 performed in flesh and skin of berries sampled from both 1103P/CS (solid bars) and M4/CS (empty bars) graft combination in 2011 growing season. Results are shown as means and SE for two biological replicates. Bars indicate SE. Different letters indicate statistically significant differences (P = 0.05) by Duncan's new multiple range test. [file Image3.TIF]

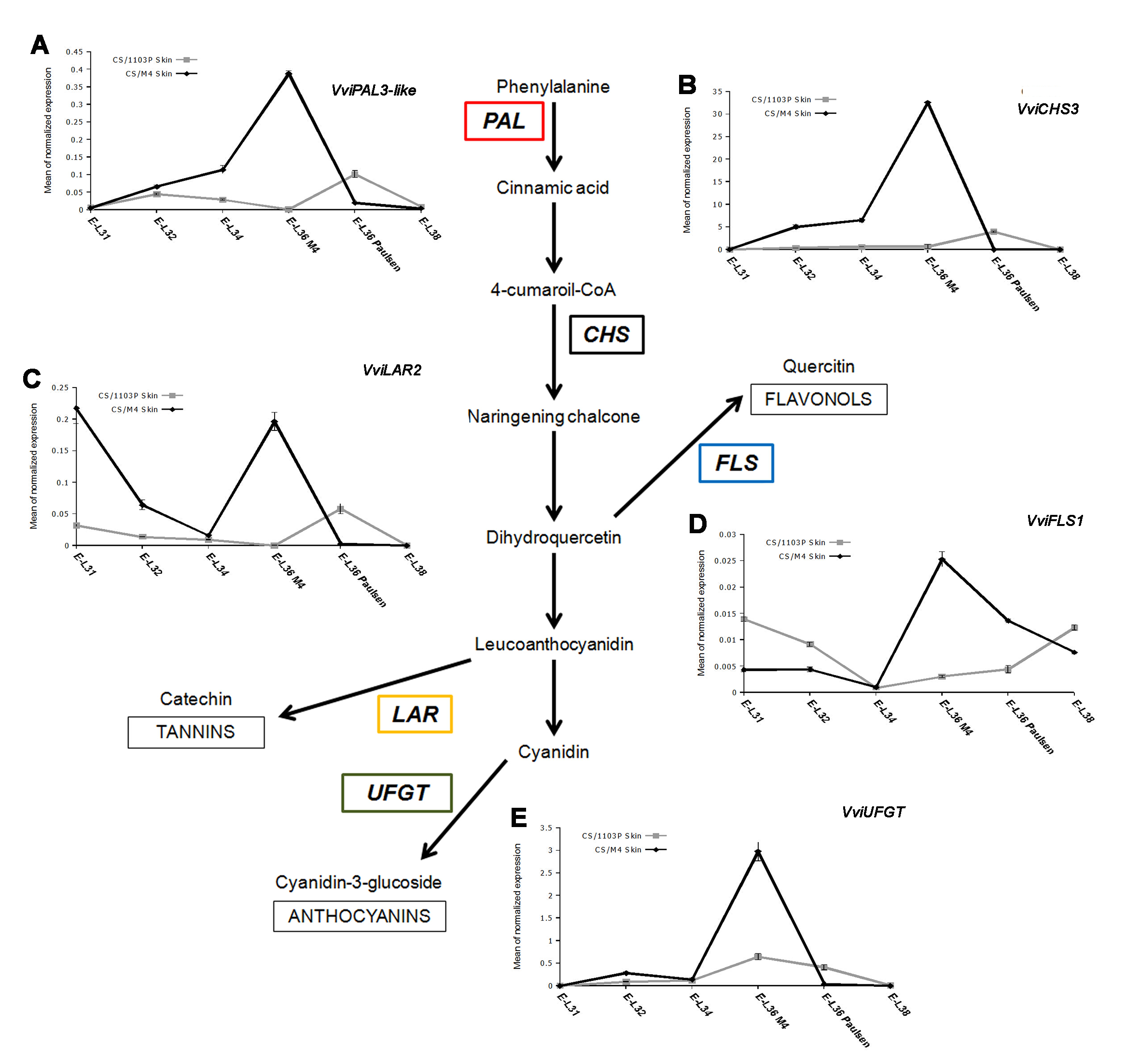

Supplement: Supplementary Figure S4 — Quantitative RT-PCR analyses on the following flavonoid-related genes: VviPAL3-like (VIT_13s0019g04460, A), VviCHS3 (VIT_05s0136g00260, B), VviLAR2 (VIT_17s0000g04150, C), VviFLS1 (VIT_18s0001g03430, D), and VviUFGT (VIT_16s0039g02230, E). Transcript levels in CS/1103P (black) and CS/M4 (white) berries are shown as means of normalized expression ±SD. [file Image4.tif]
