# Supplementary material for: Grapevine Rootstocks Differentially Affect the Rate of Ripening and Modulate Auxin-Related Genes in Cabernet Sauvignon Berries
Source: Front Plant Sci. 2016 Feb 9;7:69. doi: 10.3389/fpls.2016.00069 (PMC4746306; doi:10.3389/fpls.2016.00069)

**Supplementary Results S1**

The screening of the 12X V1 PN40024 proteome based on auxin-related proteins Pfam models led to the identification of 9 *GH3*, 10 *PIN*, 10 *PIN*-like, 21 *Aux/IAA* and 19 *ARF* genes. We first comprehensively revised the entire gene families by renaming all genes based on the nomenclature system proposed by Grimplet et al. (2014) and compared them to the corresponding families in *Arabidopsis thaliana*. Auxin related genes identified in grapevine are listed in **Table A**, together with the ORF length, the chromosomal start and stop position and the closest GenBank accession.

**List of *AUX/IAA*, *ARF*, *GH3* and *PIN* family members identified in the V1 12X grapevine annotation.** NCBI identifiers, genes localization (chromosome, start and stop), deduced protein length and previous annotation (V0 8X annotation) are provided.

| **Proposed**  **nomenclature** | **12X V1 ID** | **NCBI RefSeq** | **Chr** | **start** | **stop** | **prot**  **length** | **previous**  **nomenclature** |
| --- | --- | --- | --- | --- | --- | --- | --- |
| ***AUX/IAA*** |  |  |  |  |  |  |  |
|  |  |  |  |  |  |  |  |
| ***VviIAA15a*** | VIT_09s0002g05160 | XP_002280524 | chr9 | 4853689 | 4862025 | 238 | *VvAux/IAA12* |
| ***VviIAA15b*** | VIT_11s0016g04490 | XP_002284861 | chr11 | 3794479 | 3798406 | 184 | *VvAux/IAA8* |
| ***VviIAA29*** | VIT_04s0008g05560 | XP_002282711 | chr4 | 5035893 | 5039384 | 320 | *VvAux/IAA25* |
| ***VviIAA31*** | VIT_05s0020g01070 | XP_002275515 | chr5 | 2845633 | 2846772 | 199 |  |
| ***VviIAA35*** | VIT_05s0020g04690 | XP_002284133 | chr5 | 6548370 | 6550556 | 321 |  |
| ***VviIAA36*** | VIT_14s0081g00010 | XP_002264963 | chr14 | 7563321 | 7565613 | 170 |  |
| ***VviIAA37*** | VIT_07s0141g00290 | XP_002281771 | chr7 | 180265 | 182899 | 192 | *VvAux/IAA1* |
| ***VviIAA38*** | VIT_11s0016g03540 | XP_002284118 | chr11 | 2881444 | 2885216 | 202 |  |
| ***VviIAA39*** | VIT_09s0002g04080 | XP_002284282 | chr9 | 3749950 | 3753753 | 410 | *VvAux/IAA11* |
| ***VviIAA40*** | VIT_18s0001g08090 | XP_002285589 | chr18 | 6527840 | 6532323 | 294 | *VvAux/IAA4* |
| ***VviIAA41*** | VIT_07s0141g00270 | XP_002281696 | chr7 | 165533 | 166671 | 186 |  |
| ***VviIAA42*** | VIT_05s0020g04680 | XP_002284121 | chr5 | 6536583 | 6537443 | 168 |  |
| ***VviIAA43*** | VIT_14s0030g02310 | XP_002279955 | chr14 | 7536347 | 7537679 | 198 |  |
| ***VviIAA44*** | VIT_09s0002g05150 | XP_003632865 | chr9 | 4851013 | 4853138 | 227 |  |
| ***VviIAA45*** | VIT_07s0005g02550 | XP_002281145 | chr7 | 4884903 | 4889305 | 243 |  |
| ***VviIAA46*** | VIT_04s0008g00220 | XP_002277798 | chr4 | 162909 | 165200 | 320 |  |
| ***VviIAA47*** | VIT_09s0002g03410 | XP_002283588 | chr9 | 3075730 | 3079091 | 345 | *VvAux/IAA10* |
| ***VviIAA48*** | VIT_07s0005g04380 | XP_002269922 | chr7 | 7467838 | 7475020 | 216 |  |
| ***VviIAA49*** | VIT_05s0049g01970 | XP_002285483 | chr5 | 9458733 | 9461668 | 306 | *VvAux/IAA22* |
| ***VviIAA50*** | VIT_01s0011g04070 | XP_002279870 | chr1 | 3720067 | 3721588 | 439 |  |
| ***VviIAA51*** | VIT_11s0016g05640 | XP_002285354 | chr11 | 5095999 | 5097491 | 175 |  |
| ***ARF\*** |  |  |  |  |  |  |  |
|  |  |  |  |  |  |  |  |
| ***VviARF6a*** | VIT_12s0028g01170 | XP_002282830 | chr12 | 1746684 | 1765641 | 927 | *VvARF12* |
| ***VviARF6b*** | VIT_10s0003g04100 | XP_002279808 | chr10 | 6956492 | 6964165 | 908 | *VvARF9* |
| ***VviARF6c*** | VIT_04s0079g00200 | XP_002266678 | chr4 | 10432267 | 1E+07 | 707 | *VvARF3* |
| ***VviARF16a*** | VIT_13s0019g04380 | XP_002273590 | chr13 | 5744428 | 5747932 | 679 | *VvARF14* |
| ***VviARF16b*** | VIT_08s0040g01810 | XP_002281486 | chr8 | 12924098 | 1.3E+07 | 623 | *VvARF7* |
| ***VviARF16c*** | VIT_06s0004g02750 | XP_002282437 | chr6 | 3443777 | 3447902 | 711 | *VvARF4* |
| ***VviARF17*** | VIT_18s0001g04180 | XP_002284328 | chr18 | 3749619 | 3757678 | 347 | *VvARF17* |
| ***VviARF24*** | VIT_10s0003g00420 | XP_002273401 | chr10 | 1695104 | 1704519 | 779 | *VvARF8* |
| ***VviARF25*** | VIT_06s0004g03130 | XP_002285019 | chr6 | 3879617 | 3888296 | 798 | *VvARF5* |
| ***VviARF26*** | VIT_15s0046g00290 | XP_002264072 | chr15 | 17300361 | 1.7E+07 | 702 | *VvARF15* |
| ***VviARF27*** | VIT_02s0025g01740 | XP_002265162 | chr2 | 1653577 | 1657782 | 682 | *VvARF2* |
| ***VviARF28*** | VIT_07s0104g01230 | CBI32272 | chr7 | 2256656 | 2262296 | 696 | *VvARF6* |
| ***VviARF29*** | VIT_17s0000g00320 | XP_002284543 | chr17 | 22486 | 230745 | 862 | *VvARF16* |
| ***VviARF30*** | VIT_12s0035g01800 | XP_002268348 | chr12 | 21940164 | 2.2E+07 | 678 | *VvARF13* |
| ***VviARF31*** | VIT_18s0089g00910 | XP_002266947 | chr18 | 28749580 | 2.9E+07 | 649 | *VvARF19* |
| ***VviARF32*** | VIT_01s0244g00150 | XP_002268849 | chr1 | 21717673 | 2.2E+07 | 769 | *VvARF1* |
| ***VviARF33*** | VIT_11s0016g00640 | CAN69277 | chr11 | 629393 | 640201 | 1155 | *VvARF10* |
| ***VviARF34*** | VIT_18s0001g13930 | XP_002266947 | chr18 | 11920505 | 1.2E+07 | 953 | *VvARF18* |
| ***VviARF35*** | VIT_11s0065g00310 | XP_002266603 | chr11 | 13985443 | 1.4E+07 | 1115 | *VvARF11* |
|  |  |  |  |  |  |  |  |
| ***GH3*** |  |  |  |  |  |  |  |
|  |  |  |  |  |  |  |  |
| ***VviGH3-9*** | VIT_07s0005g00090 | XP_002263353 | chr7 | 2895937 | 2898369 | 596 | *VvGH3-4* |
| ***VviGH3-10*** | VIT_12s0134g00230 | XP_002276241 | chr12 | 7753336 | 7755364 | 614 | *VvGH3-5* |
| ***VviGH3-17*** | VIT_12s0059g01870 | XP_002272560 | chr12 | 6726733 | 6729340 | 592 | *VvGH3-7* |
| ***VviGH3-21*** | VIT_07s0104g00800 | XP_002271002 | chr7 | 1956032 | 1958349 | 578 | *VvGH3-8* |
| ***VviGH3-22*** | VIT_07s0129g00660 | XP_002283886 | chr7 | 15895858 | 1.6E+07 | 600 | *VvGH3-2* |
| ***VviGH3-23*** | VIT_03s0091g00310 | XP_002271252 | chr3 | 6633708 | 6635996 | 598 | *VvGH3-1* |
| ***VviGH3-24*** | VIT_01s0150g00300 | XP_002268278 | chr1 | 22706570 | 2.3E+07 | 591 | *VvGH3-6* |
| ***VviGH3-25*** | VIT_15s0046g01280 | XP_002280738 | chr15 | 18284929 | 1.8E+07 | 648 | *VvGH3-9* |
| ***VviGH3-26*** | VIT_19s0014g04690 | XP_002283229 | chr19 | 5016103 | 5018391 | 613 | *VvGH3-3* |
|  |  |  |  |  |  |  |  |
|  |  |  |  |  |  |  |  |
|  |  |  |  |  |  |  |  |
|  |  |  |  |  |  |  |  |
|  |  |  |  |  |  |  |  |
|  |  |  |  |  |  |  |  |
|  |  |  |  |  |  |  |  |
|  |  |  |  |  |  |  |  |
|  |  |  |  |  |  |  |  |

**Phylogenetic relationship in grapevine, arabidopsis and tomato auxin-related deduced proteins.**

**A.** **Phylogenetic relationship of ARF proteins.** Multiple sequence alignment for full-length transcription factors was carried out using MUSCLE. The evolutionary history was inferred using the Maximum Likelihood method based on the JTT matrix-based model. The bootstrap consensus tree was inferred from 100 bootstrap replicates. Branches corresponding to partitions reproduced in less than 70% bootstrap replicates are collapsed. The percentage of replicate trees (lower that 70%) in which the associated taxa clustered together in the bootstrap test is shown next to the branches. Evolutionary analyses were conducted in MEGA6.


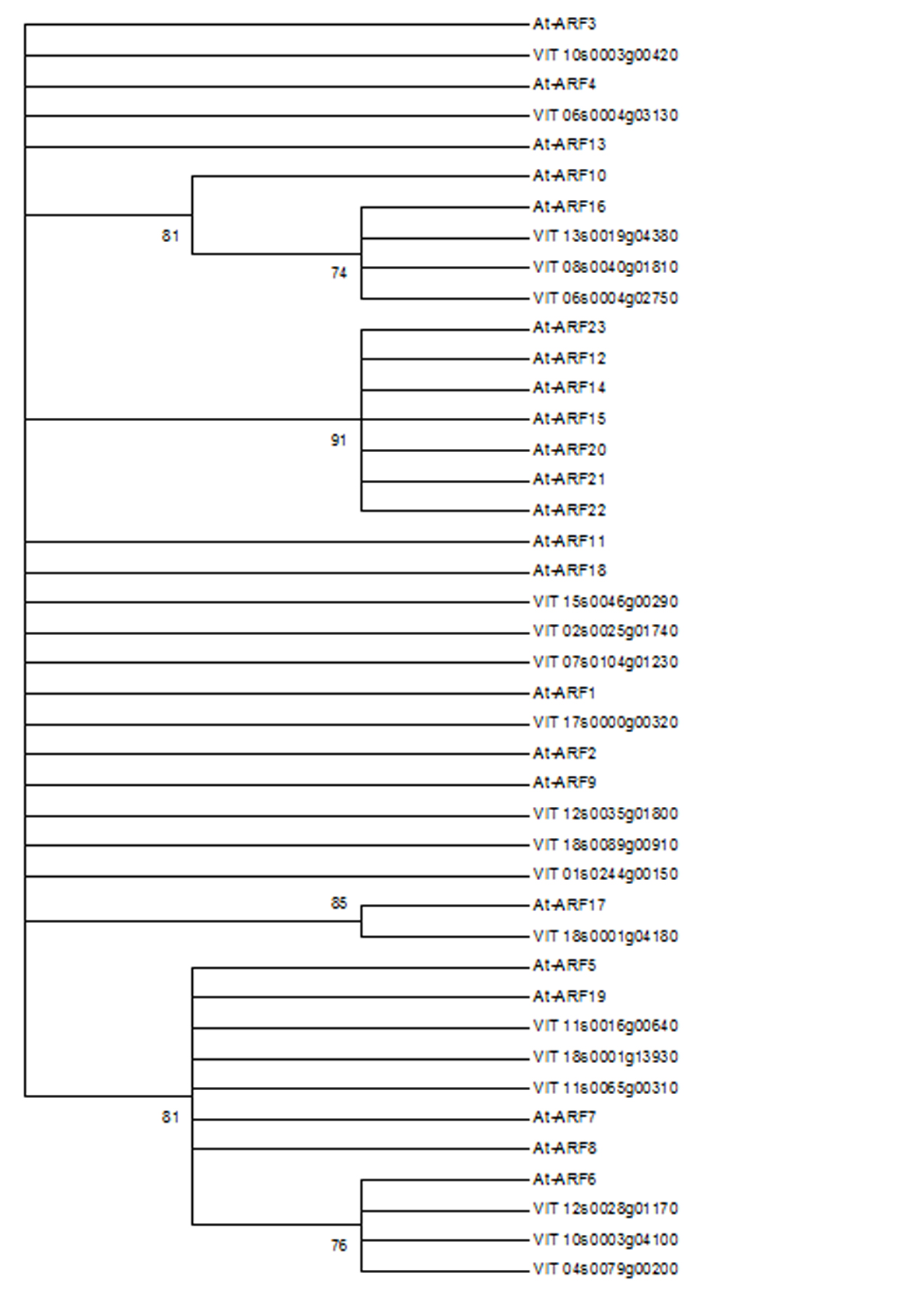


**B. Phylogenetic relationship of IAA proteins.** Multiple sequence alignment for full-length transcription factors was carried out using MUSCLE. The evolutionary history was inferred using the Maximum Likelihood method based on the JTT matrix-based model. The bootstrap consensus tree was inferred from 100 bootstrap replicates. Branches corresponding to partitions reproduced in less than 70% bootstrap replicates are collapsed. The percentage of replicate trees (lower that 70%) in which the associated taxa clustered together in the bootstrap test is shown next to the branches. Evolutionary analyses were conducted in MEGA6.


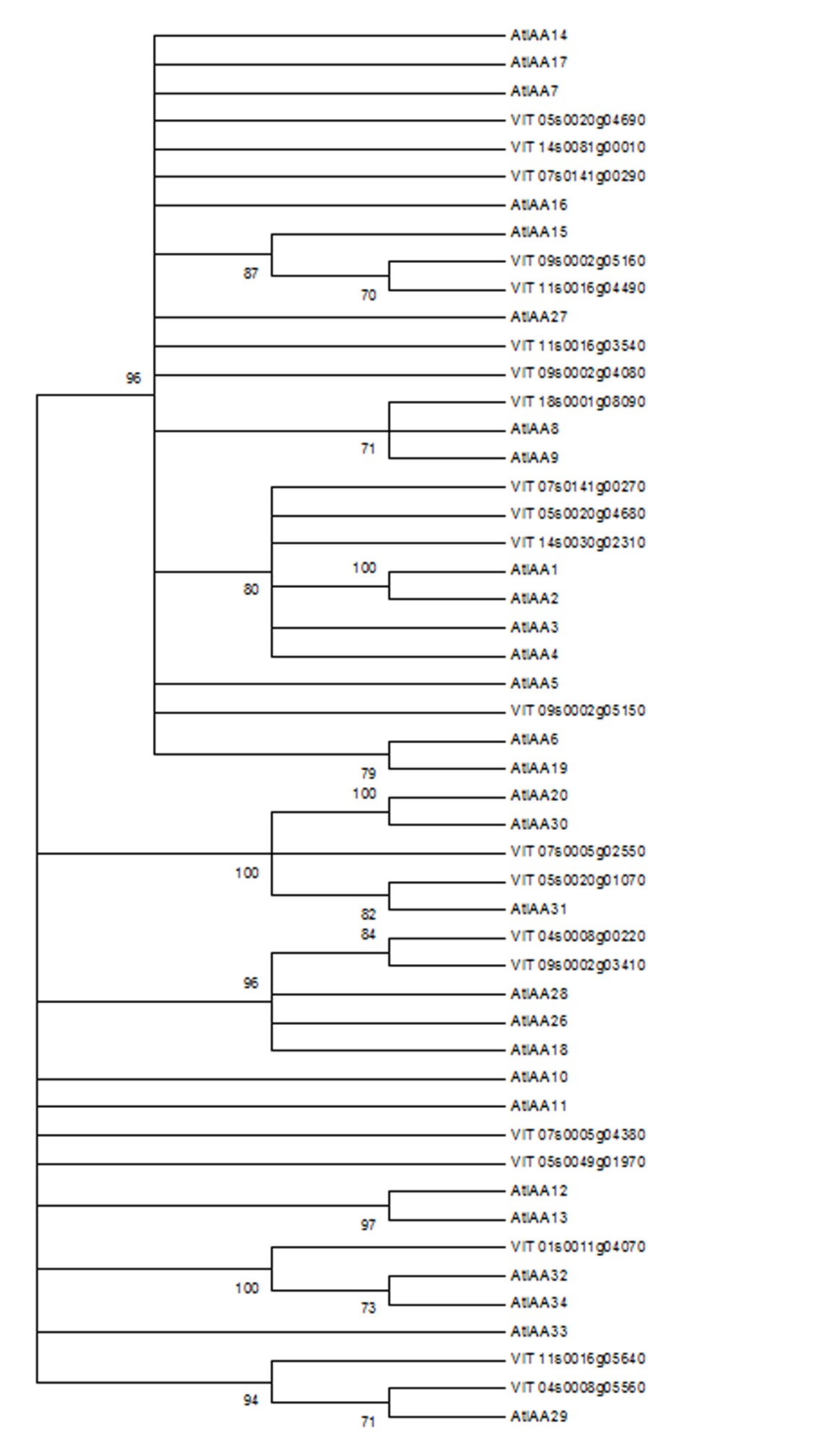


**D. Phylogenetic relationship of GH3 proteins.** Multiple sequence alignment for full-length transcription factors was carried out using MUSCLE. The evolutionary history was inferred using the Maximum Likelihood method based on the JTT matrix-based model. The bootstrap consensus tree was inferred from 100 bootstrap replicates. Branches corresponding to partitions reproduced in less than 70% bootstrap replicates are collapsed. The percentage of replicate trees (lower that 70%) in which the associated taxa clustered together in the bootstrap test is shown next to the branches. Evolutionary analyses were conducted in MEGA6.


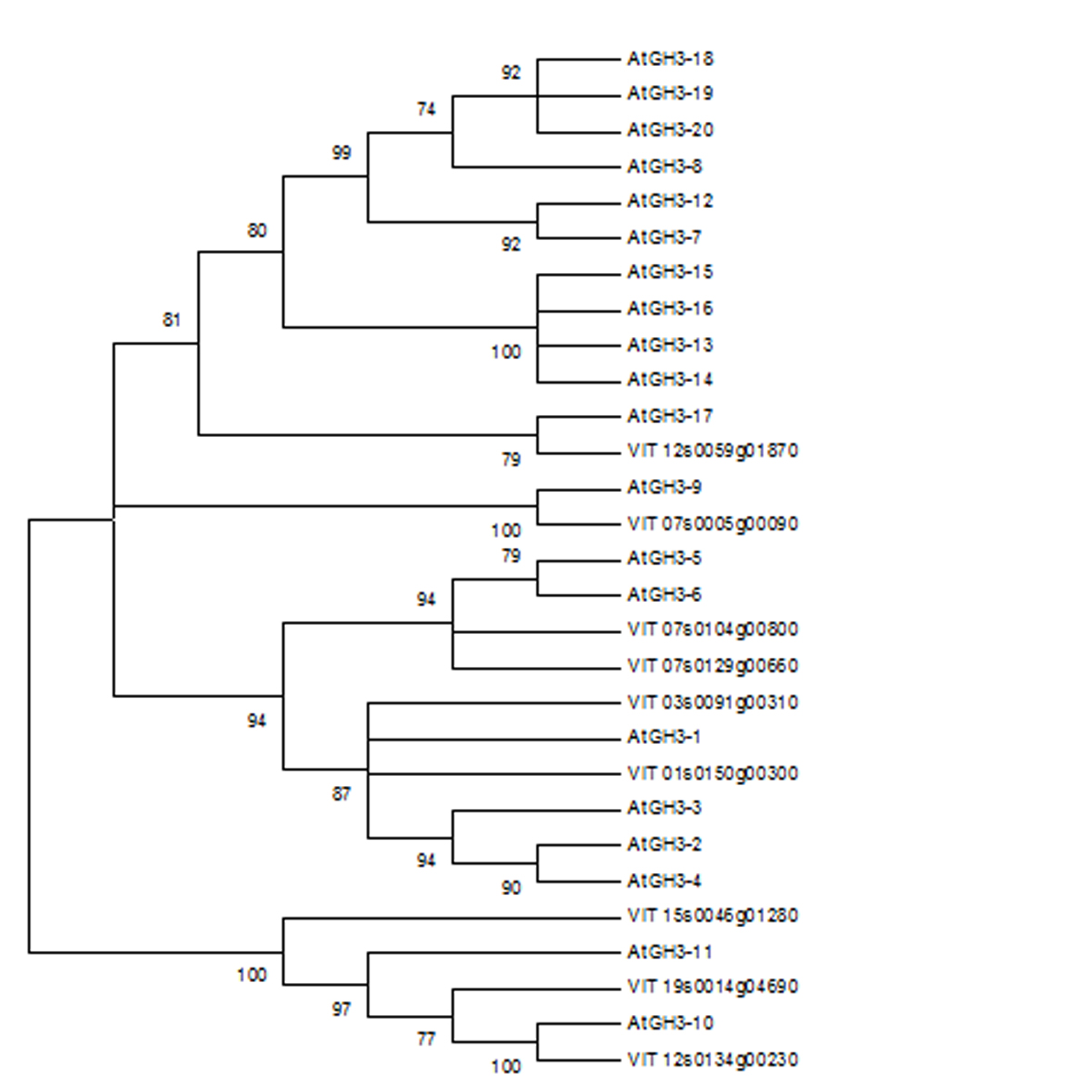

Supplement: Supplementary Results S1 — Nomenclature of genes belonging to the grapevine ARF, Aux/IAA, and GH3 multigenic families. [file SupplementaryResultsS1.DOCX]
